# Taurochenodeoxycholic acid alleviates obesity-induced endothelial dysfunction

**DOI:** 10.1093/eurheartj/ehaf766

**Published:** 2025-10-03

**Authors:** Hanlin Lu, Zhinan Wu, Meng Wan, Sisi Xiong, Xin Huang, Teng Liu, Xiuxin Jiang, Lifan He, Chang Ma, Huiliang Cui, Xiaolin Yue, Jingyuan Li, Xiaoteng Qin, Yawei Wang, Cheng Zhang, Jianmin Yang, Shaozhuang Liu, Wencheng Zhang

**Affiliations:** State Key Laboratory for Innovation and Transformation of Luobing Theory, Key Laboratory of Cardiovascular Remodeling and Function Research of MOE, NHC, CAMS and Shandong Province, Department of Cardiology, Qilu Hospital of Shandong University, No. 107, Wen Hua Xi Rd, Jinan 250012, China; State Key Laboratory for Innovation and Transformation of Luobing Theory, Key Laboratory of Cardiovascular Remodeling and Function Research of MOE, NHC, CAMS and Shandong Province, Department of Cardiology, Qilu Hospital of Shandong University, No. 107, Wen Hua Xi Rd, Jinan 250012, China; Department of Gastroenterology, Qilu Hospital of Shandong University, Jinan, China; Division of Bariatric and Metabolic Surgery, Department of General Surgery, Qilu Hospital of Shandong University, Jinan, China; Division of Bariatric and Metabolic Surgery, Department of General Surgery, Qilu Hospital of Shandong University, Jinan, China; Division of Bariatric and Metabolic Surgery, Department of General Surgery, Qilu Hospital of Shandong University, Jinan, China; Division of Bariatric and Metabolic Surgery, Department of General Surgery, Qilu Hospital of Shandong University, Jinan, China; State Key Laboratory for Innovation and Transformation of Luobing Theory, Key Laboratory of Cardiovascular Remodeling and Function Research of MOE, NHC, CAMS and Shandong Province, Department of Cardiology, Qilu Hospital of Shandong University, No. 107, Wen Hua Xi Rd, Jinan 250012, China; State Key Laboratory for Innovation and Transformation of Luobing Theory, Key Laboratory of Cardiovascular Remodeling and Function Research of MOE, NHC, CAMS and Shandong Province, Department of Cardiology, Qilu Hospital of Shandong University, No. 107, Wen Hua Xi Rd, Jinan 250012, China; State Key Laboratory for Innovation and Transformation of Luobing Theory, Key Laboratory of Cardiovascular Remodeling and Function Research of MOE, NHC, CAMS and Shandong Province, Department of Cardiology, Qilu Hospital of Shandong University, No. 107, Wen Hua Xi Rd, Jinan 250012, China; State Key Laboratory for Innovation and Transformation of Luobing Theory, Key Laboratory of Cardiovascular Remodeling and Function Research of MOE, NHC, CAMS and Shandong Province, Department of Cardiology, Qilu Hospital of Shandong University, No. 107, Wen Hua Xi Rd, Jinan 250012, China; State Key Laboratory for Innovation and Transformation of Luobing Theory, Key Laboratory of Cardiovascular Remodeling and Function Research of MOE, NHC, CAMS and Shandong Province, Department of Cardiology, Qilu Hospital of Shandong University, No. 107, Wen Hua Xi Rd, Jinan 250012, China; State Key Laboratory for Innovation and Transformation of Luobing Theory, Key Laboratory of Cardiovascular Remodeling and Function Research of MOE, NHC, CAMS and Shandong Province, Department of Cardiology, Qilu Hospital of Shandong University, No. 107, Wen Hua Xi Rd, Jinan 250012, China; State Key Laboratory for Innovation and Transformation of Luobing Theory, Key Laboratory of Cardiovascular Remodeling and Function Research of MOE, NHC, CAMS and Shandong Province, Department of Cardiology, Qilu Hospital of Shandong University, No. 107, Wen Hua Xi Rd, Jinan 250012, China; State Key Laboratory for Innovation and Transformation of Luobing Theory, Key Laboratory of Cardiovascular Remodeling and Function Research of MOE, NHC, CAMS and Shandong Province, Department of Cardiology, Qilu Hospital of Shandong University, No. 107, Wen Hua Xi Rd, Jinan 250012, China; State Key Laboratory for Innovation and Transformation of Luobing Theory, Key Laboratory of Cardiovascular Remodeling and Function Research of MOE, NHC, CAMS and Shandong Province, Department of Cardiology, Qilu Hospital of Shandong University, No. 107, Wen Hua Xi Rd, Jinan 250012, China; Division of Bariatric and Metabolic Surgery, Department of General Surgery, Qilu Hospital of Shandong University, Jinan, China; State Key Laboratory for Innovation and Transformation of Luobing Theory, Key Laboratory of Cardiovascular Remodeling and Function Research of MOE, NHC, CAMS and Shandong Province, Department of Cardiology, Qilu Hospital of Shandong University, No. 107, Wen Hua Xi Rd, Jinan 250012, China

**Keywords:** Taurochenodeoxycholic acid, Farnesoid X receptor, Obesity, Endothelial dysfunction, Hypertension, Serine

## Abstract

**Background and Aims:**

Obesity is a global health challenge significantly increasing cardiovascular disease (CVD) burden. Effective prevention and treatment necessitate targeting early pathological changes, particularly obesity-induced endothelial dysfunction (ED). This study aimed to characterize ED heterogeneity in non-hypertensive obese (NHO) individuals, investigate the association of serum metabolites with obesity-induced ED, and identify potentially predictive and therapeutic metabolites.

**Methods:**

Utilizing wire myograph, this study assessed ED of *ex vivo* arterioles from omental adipose tissue of 213 NHO patients, categorized into metabolically healthy obesity (MHO) and metabolically unhealthy obesity (MUO). Targeted metabolomic profiling identified associations between serum metabolites and ED.

**Results:**

Obesity-induced ED in NHO patients lacked correlations with many traditional cardiovascular risk factors. The MHO and MUO individuals exhibited similar ED and metabolomic profile characteristics. Serum metabolomics identified bile acids (BAs), particularly chenodeoxycholic acid (CDCA), as negatively correlated with ED in NHO patients. Taurochenodeoxycholic acid (TCDCA), a taurine-conjugated derivative of CDCA, protected against obesity-induced ED and hypertension. Mechanistically, endothelial Farnesoid X receptor (FXR) deletion aggravated obesity-induced ED and hypertension, negating the beneficial effects of bariatric surgery or TCDCA treatment. The TCDCA-FXR activation in endothelial cells upregulated ATF4 transcription, which was suppressed by PHB1, thereby enhancing serine and one-carbon metabolism.

**Conclusions:**

This study suggests CDCA as a promising biomarker for identification of obesity-induced ED. Taurochenodeoxycholic acid demonstrates significant therapeutic potential for alleviating various forms of obesity-induced ED. This effect is mediated by the endothelial TCDCA-FXR-PHB1-ATF4 axis, which upregulates serine and one-carbon metabolism, thereby offering a novel strategy to delay the onset of hypertension and other CVDs.

## Structured Graphical Abstract

**Structured Graphical Abstract:**
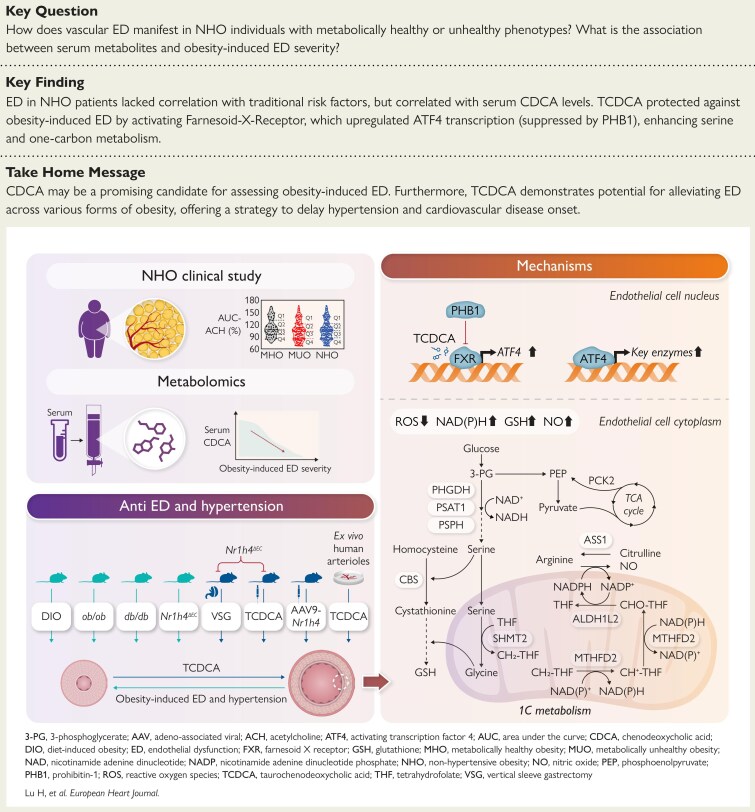
Utilizing wire myograph, this study assessed ED of *ex vivo* arterioles from omental adipose tissue of 213 NHO patients, categorized into MHO and MUO. Serum metabolomics identified CDCA as negatively correlated with ED in NHO patients. TCDCA, a taurine-conjugated derivative of CDCA, protected against obesity-induced ED and hypertension. Mechanistically, endothelial FXR deletion aggravated obesity-induced ED and hypertension, negating the beneficial effects of bariatric surgery or TCDCA treatment. TCDCA-FXR activation in endothelial cells upregulated ATF4 transcription, which was suppressed by PHB1, thereby enhancing serine and one-carbon metabolism.

**See the editorial comment for this article ‘From bile to vessels: linking obesity to endothelial dysfunction’, by S. Kraler**  ***et al*****., https://doi.org/10.1093/eurheartj/ehaf957.**

Translational perspective Critical for preventing cardiovascular disease in obese individuals is identifying those at highest risk. Findings from the assessment of arterioles from 213 non-hypertensive obese patients using wire myography revealed considerable endothelial dysfunction (ED) heterogeneity, a condition not accurately predicted by traditional cardiovascular risk factors. Serum metabolomics identifies chenodeoxycholic acid as a potential marker for assessing obesity-induced ED, even in apparently metabolically healthy individuals. Taurochenodeoxycholic acid protected against obesity-induced ED and hypertension in various models through FXR activation, which upregulated ATF4 (suppressed by PHB1), enhancing serine and one-carbon metabolism. These findings offer new avenues for risk prediction and developing treatments.

## Introduction

Obesity, a complex, epidemic disease of excessive adiposity, significantly elevates risk for conditions like cardiovascular disease (CVD) and metabolic syndrome.^1–3^ Interestingly, not all individuals meeting the criteria for obesity develop metabolic complications. Metabolic heterogeneity exists within obese populations, yielding phenotypes like metabolically healthy obesity (MHO) and metabolically unhealthy obesity (MUO).^4,5^ Despite appearing initially free of cardiometabolic risk factors,^6^ MHO individuals face a significant (∼50%) higher CVD risk compared with metabolically healthy non-obese controls.^7^ This heightened risk is strongly associated with endothelial dysfunction (ED), now recognized as a critical early therapeutic target for obesity-related CVD.^8,9^ Crucially, the impact of serum metabolomics on ED heterogeneity in obesity remains largely unexplored.

Bile acids (BAs) function as key metabolic regulators via enterohepatic and systemic signalling,^10^ influencing obesity-related diseases even at low circulating levels.^11^ The nuclear receptor Farnesoid X receptor (FXR), expressed in endothelial cells (ECs) and implicated in CVD, mediates some BA effects.^12–14^ While circulating BAs interact directly with ECs, their specific impact, particularly in human vessels and relevant models, requires further investigation.^15^

Here, we investigated vascular phenotypes and serum metabolic profiles in MHO and MUO individuals. We identified a unique BA signature, highlighted by chenodeoxycholic acid (CDCA), correlating with obesity-induced ED. We further evaluated various BAs, focusing on taurochenodeoxycholic acid (TCDCA), for their potential to mitigate obesity-induced ED using *ex vivo* human arterioles and multiple models. We elucidated mechanisms underlying TCDCA-FXR-ATF4 signalling in ED, specifically examining transcriptional regulation and serine/one-carbon metabolism.^16^ Our findings uncover mechanisms driving endothelial functional heterogeneity in obesity, offering potential for earlier identification of severe ED and targeted therapeutic strategies for obesity-induced CVD.

## Methods

Detailed methods are provided in the Supplementary data.

## Results

### *Ex vivo* vascular characterization of patients with non-hypertensive obesity

**Figure 1:**
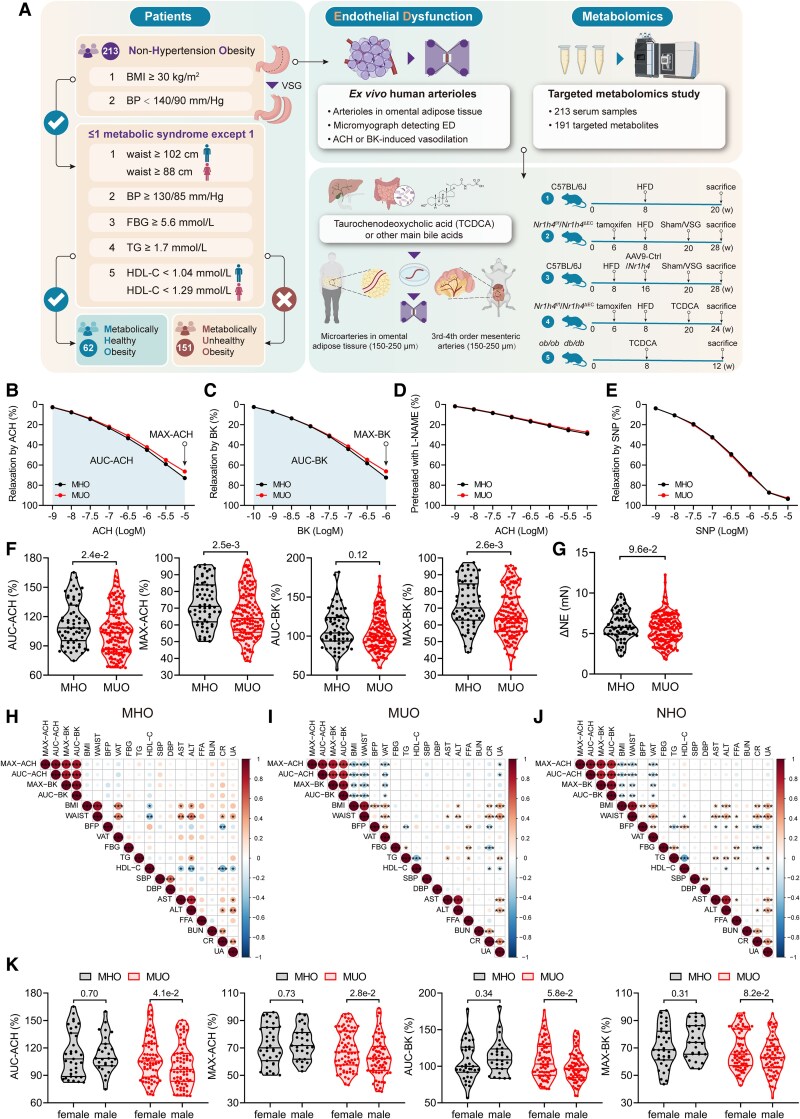
*Ex vivo* vascular characterization of patients with non-hypertensive obesity. (*A*) Human study and experiment flow chart: all the patients with non-hypertensive obese (*n* = 213) including the patients with metabolically healthy obesity (*n* = 62) and with metabolically unhealthy obesity (*n* = 151). (*B* and *C*) Endothelium-dependent vasodilation to cumulative concentration of acetylcholine (*B*) or bradykinin (*C*) in *ex vivo* human arterioles in the metabolically healthy obesity (*n* = 62) and metabolically unhealthy obesity groups (*n* = 151). AUC-ACH, area under the curve of ACH-induced response; MAX-ACH, peak amplitude of 10^−5^ M ACH response; AUC-BK, area under the curve of BK-induced response; MAX-BK, peak amplitude of 10^−6^ M BK response. (*D* and *E*) Endothelium-independent vasodilation to cumulative concentration of acetylcholine in *ex vivo* human arterioles pre-treated with L-NAME (*D*) or to cumulative concentration of sodium nitroprusside (*E*) in the metabolically healthy obesity (*n* = 62) and metabolically unhealthy obesity groups (*n* = 151). (*F*) AUC-ACH, MAX-ACH, AUC-BK, and MAX-BK in the metabolically healthy obesity (*n* = 62) and metabolically unhealthy obesity groups (*n* = 151). (*G*) Contraction of human arterioles inresponse to 10^-5^ NE. (*H–J*) Correlation heatmap between four endothelial dysfunction metrics (AUC-ACH, MAX-ACH, AUC-BK, MAX-BK) and clinical characteristics in the metabolically healthy obesity group (*H*, *n* = 62), the metabolically unhealthy obesity group (*I*, *n* = 151), or the non-hypertensive obese population (*J*, *n* = 213). BMI, body mass index; BFP, body fat percentage; VAT, visceral adipose tissue area; FBG, fasting blood glucose; TG, triglyceride; HDL-C, HDL cholesterol; SBP, systolic blood pressure; DBP, diastolic blood pressure; AST, aspartate aminotransferase; ALT, alanine aminotransferase; FFA, free fatty acids; BUN, blood urea nitrogen; CR, creatinine; UA, uric acid. (*K*) AUC-ACH, MAX-ACH, AUC-BK, and MAX-BK of female or male individuals separately in the metabolically healthy obesity and metabolically unhealthy obesity groups. Data are presented as median with interquartile range. Statistical analysis was performed using Mann–Whitney *U* test (*F, G* and *K*) and Spearman correlation (*H–J*) with Benjamini–Hochberg correction for multiple comparisons to control false discovery rate. *Adjusted *P* < .05; **adjusted *P* < .01; ***adjusted *P* < .001

To investigate early-stage ED in obesity, we recruited 213 non-hypertensive obese (NHO) individuals based on specific criteria (*Figure 1A*). Metabolically healthy obesity was defined as obesity with no more than one of the components of metabolic syndrome (excluding increased waist circumference): high blood pressure (BP), high fasting blood glucose (FBG), high triglyceride (TG), and low HDL cholesterol (HDL-C) concentration.^17–19^ The cohort comprised 62 MHO participants and 151 MUO participants. Baseline clinical characteristics are detailed in Supplementary data online, *Table S1*.

Arterioles (150–250 µm) were isolated from omental adipose tissue during vertical sleeve gastrectomy (VSG) and assessed for vasodilation using wire myograph (*Figure 1A* and Supplementary data online, *Figure S1*). Endothelium-dependent vasodilation was determined by acetylcholine (ACH) or bradykinin (BK) inducing responses, while endothelium-independent function was assessed via ACH with L-NAME pre-treatment or sodium nitroprusside (SNP) inducing vasodilation (*Figure 1B–E*). Endothelial dysfunction severity was quantified using four metrics: area under the curve (AUC) and maximum response (MAX) for both ACH and BK (AUC-ACH, MAX-ACH, AUC-BK, MAX-BK). As shown in *Figure 1F*, the MUO group exhibited significantly more severe ED compared with the MHO group. No significant difference in contraction function was observed between the MHO and MUO groups (*Figure 1G*).

Analysing correlations between ED metrics and clinical characteristics revealed striking differences between groups (*Figure 1H–J*). While endothelium-dependent vasodilation in MHO patients showed no significant correlation with traditional obesity measures [body mass index (BMI), waist circumference, body fat percentage (BFP), visceral adipose tissue (VAT) area] or metabolic syndrome components (FBG, TG, HDL-C, SBP, DBP), a significant negative correlation with BMI, waist circumference, and VAT area was observed within the MUO group. Despite these subgroup-specific associations, interaction analysis (see Supplementary data online, *Table S2*) indicated that metabolic health status (MHO vs MUO) did not significantly alter the relationship between ED (quantified by AUC-ACH level) and traditional cardiovascular risk factors. Although a significant increase in ED with escalating BMI, waist circumference, and VAT area was evident across the entire NHO cohort, this trend was predominantly driven by the MUO subgroup (*Figure 1J*). Gender analysis within the MUO group showed males exhibited more pronounced ED than females, a difference not observed in the MHO group (*Figure 1K*).

Non-hypertensive obese patients were categorized into MHO and MUO groups to explore differences in ED. Although *ex vivo* myograph revealed significant individual variability in ED, the characteristics of ED were broadly similar between MHO and MUO and demonstrated no strong association with many traditional cardiovascular risk factors, especially within the MHO group. These findings suggest that even ‘metabolically healthy’ individuals may not be endothelially healthy, underscoring the critical need for early cardiovascular monitoring in all obese patients and the imperative for further mechanistic investigation to facilitate timely interventions for associated CVDs.

### Metabolomics reveals bile acids, notably chenodeoxycholic acid, associated with obesity-induced endothelial dysfunction

**Figure 2:**
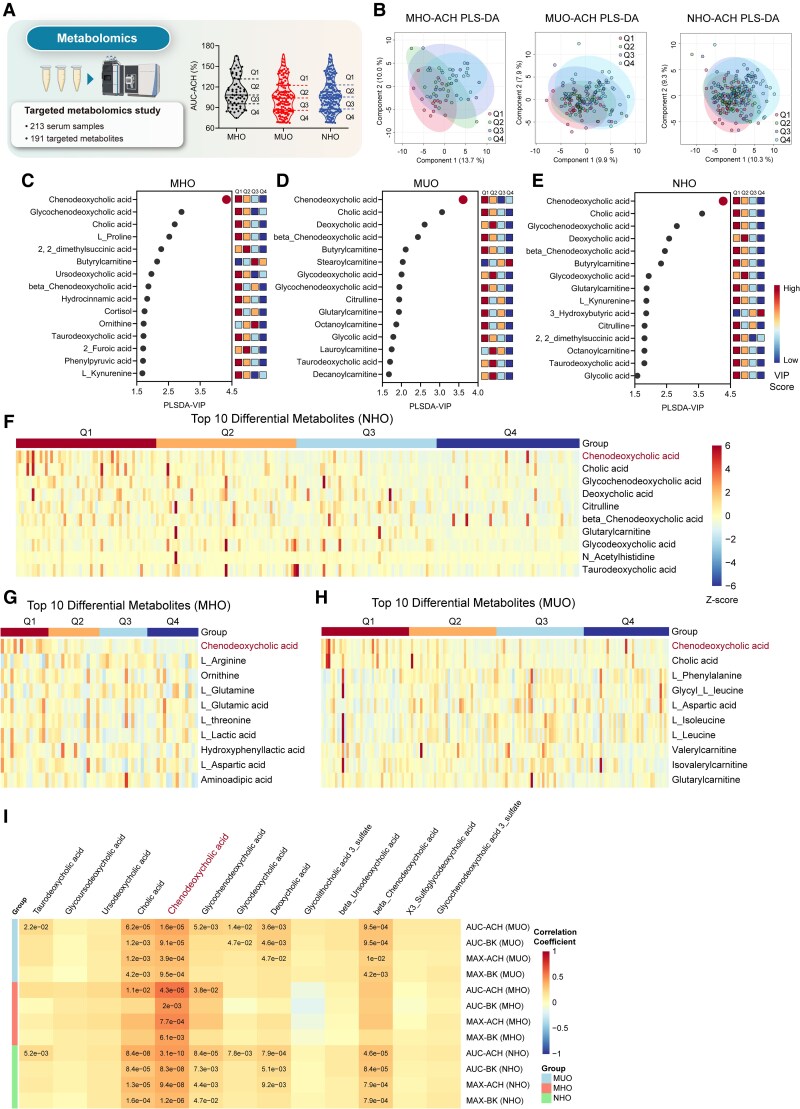
Metabolomics reveals bile acids, notably chenodeoxycholic acid, associated with obesity-induced endothelial dysfunction. (*A*) Metabolomics flow chart and schematic diagram for AUC-ACH-IQR (Q1, Q2 Q3, Q4 groups) in the metabolically healthy obesity group (*n* = 62), the metabolically unhealthy obesity group (*n* = 151), or the non-hypertensive obese group (*n* = 213). The AUC-ACH data are derived from the experiments shown in *Figure 1*. (*B*) Partial least squares discriminant analysis showing the metabolites of Q1–Q4 patients in the metabolically healthy obesity, metabolically unhealthy obesity, and non-hypertensive obese groups. (*C–E*) Variable importance in projection scores of the partial least squares discriminant analysis based on metabolic profile in the metabolically healthy obesity (*C*), metabolically unhealthy obesity (*D*), and non-hypertensive obese (*E*) groups. (*F–H*) Heatmap of top 10 differential metabolites across Q1–Q4 groups in the non-hypertensive obese (*F*), metabolically healthy obesity (*G*), and metabolically unhealthy obesity (*H*) groups. (*I*) Correlation heatmap between four endothelial dysfunction metrics (AUC-ACH, MAX-ACH, AUC-BK, MAX-BK) and 13 types of bile acids (detected in the serum metabolomics) levels in the metabolically healthy obesity group, the metabolically unhealthy obesity group, and the non-hypertensive obese group. Significant *P* values (<.05) are indicated within the heatmap cells. Data are presented as median with interquartile range. Statistical analysis was performed using the Kruskal–Wallis test with Benjamini–Hochberg correction for multiple comparisons to control false discovery rate (*F–H*) and Spearman correlation with Benjamini–Hochberg correction for multiple comparisons to control FDR (*I*)

To investigate metabolite alterations in NHO cohort, we performed targeted serum metabolomic profiling on 62 MHO and 151 MUO patients. Stratifying patients into quartiles (Q1−Q4) based on AUC-ACH values revealed a progressive decline in ED severity from Q1 (least ED) to Q4 (most ED), as illustrated in violin plots (*Figure 2A*). Immunofluorescence (IF) confirmed increased ED markers VCAM-1^20^ and endothelin-1 (ET-1)^21^ in Q4 vs Q1, while von Willebrand factor^22^ remained unchanged (see Supplementary data online, *Figure S2*). Comprehensive quantification of 191 metabolites using partial least squares discriminant analysis (PLS-DA), which demonstrated significant metabolic distinctions among Q1–Q4 groups (*Figure 2B*; Supplementary data online, *Figure S3* shows all 191 metabolite levels). Quantitative analysis highlighted BAs, particularly CDCA and its derivative glycochenodeoxycholic acid (GCDCA), exhibiting the highest variable importance in projection (VIP) scores in the MHO group, indicating strong association with ED (*Figure 2C*). Analysis in the MUO group (*Figure 2D*) and the combined NHO cohort (*Figure 2E*) similarly identified various BAs, especially CDCA and cholic acid (CA), as primary contributors associated with alleviating ED (Q4–Q1). The top 10 differential metabolites across Q1–Q4 groups are presented in a heatmap (*Figure 2F–H*), showing consistently higher CDCA levels in the Q1 group. These findings underscore CDCA’s pivotal role in driving the heterogeneity of obesity-induced ED.

Findings were validated by stratifying patients based on AUC-BK (see Supplementary data online, *Figure S4A*). The PLS-DA plots again showed clear metabolic separation (see Supplementary data online, *Figure S4B*), and VIP scores consistently identified CDCA as the top variable metabolite (see Supplementary data online, *Figure S4C*–*E*). Supplementary data online, *Figure S4F*–*H* illustrates a gradual decline in CDCA levels with worsening ED (decreasing AUC-BK). Further metabolomic profiling using orthogonal PLS-DA directly comparing Q1 and Q4 consistently identified CDCA as a top-ranking metabolite via S-plots, reinforcing its potential pivotal role (see Supplementary data online, *Figure S5A*–*F*).

Spearman correlation analysis between the four ED indicators and all 191 metabolites revealed significant associations predominantly within the BA class, particularly for CDCA and CA, with minimal comparable findings in other classes (see Supplementary data online, *Figure S6*). Focused analysis of 13 quantifiable BAs demonstrated positive correlations between CDCA levels and endothelium-dependent vasodilation across all groups (*Figure 2I*). Investigating sex-specific metabolic patterns revealed significant sex-based metabolic differences largely overlapping with metabolites discriminating ED quartiles (see Supplementary data online, *Figure S7A*–*B*), aligning with our earlier finding of more severe ED in males. Direct comparison between MHO and MUO metabolic patterns also showed marked variations in CDCA and CA (see Supplementary data online, *Figure S7C* and *D*).

Collectively, these findings strongly suggest BAs, specifically CDCA, function as critical modulators of obesity-induced ED, potentially representing a potential marker associated with obesity-induced ED.

### Taurochenodeoxycholic acid rescues obesity-induced endothelial dysfunction in *ex vivo* human arterioles

**Figure 3:**
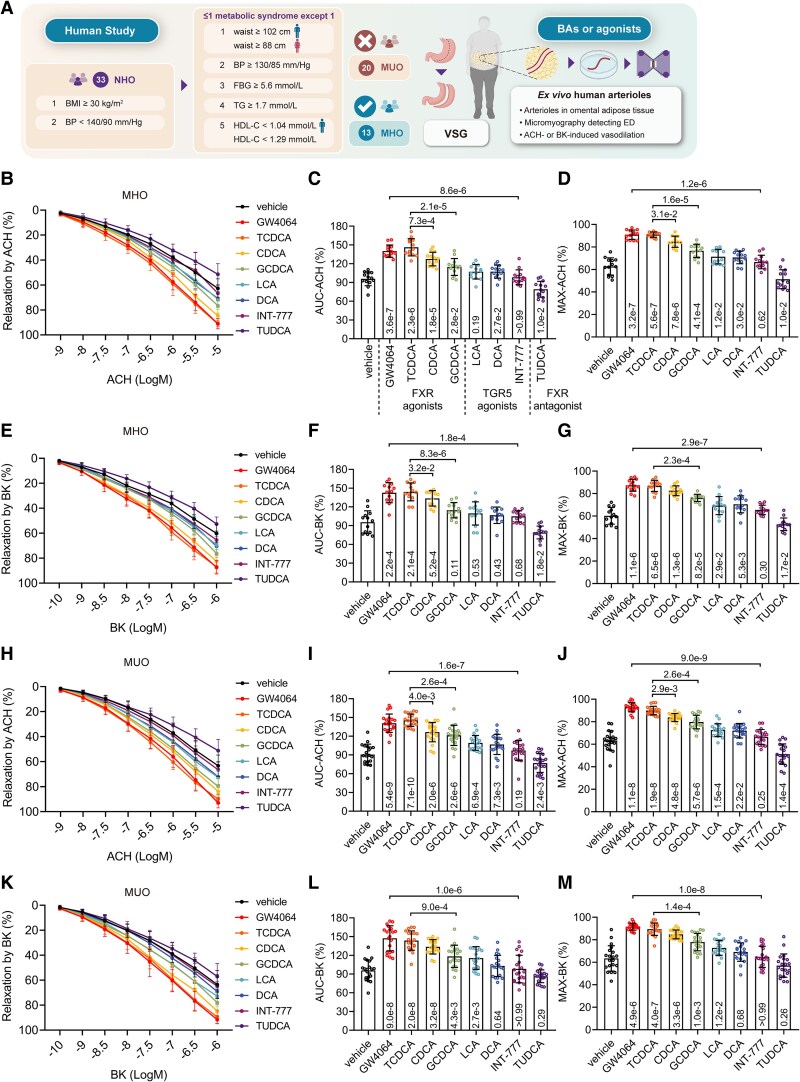
Taurochenodeoxycholic acid rescues obesity-induced endothelial dysfunction in *ex vivo* human arterioles. (*A*) Study design flow chart: non-hypertensive obese participants (*n* = 33) were stratified into metabolically healthy obesity (*n* = 13) and metabolically unhealthy obesity (*n* = 20) subgroups. Omental arteriole rings were divided into equal segments and incubated with different bile acids or agonists (GW4064 10 µM, INT-777 10 µM, TCDCA 50 µM, CDCA 50 µM, GCDCA 50 µM, DCA 50 µM, LCA 50 µM, TUDCA 50 µM) for 12 h before assessment by wire myograph. (*B–M*) Endothelium-dependent vasodilation in arterioles from: (*B–D*) metabolically healthy obesity participants showing acetylcholine-mediated vasodilation (*B*), with quantitative AUC-ACH (*C*) and MAX-ACH (*D*) analyses; (*E–G*) metabolically healthy obesity participants showing bradykinin-mediated vasodilation (*E*), with AUC-BK (*F*) and MAX-BK (*G*) analyses; (*H–J*) metabolically unhealthy obesity participants showing acetylcholine responses (*H*) with AUC-ACH (*I*) and MAX-ACH (*J*); (*K–M*) metabolically unhealthy obesity participants showing bradykinin responses (*K*) with AUC-BK (*L*) and MAX-BK (*M*). Data are presented as mean ± standard deviation. Statistical analysis was performed using repeated measures one-way ANOVA with Greenhouse–Geisser correction followed by with Tukey’s *post hoc* test

Our clinical metabolomics data revealing elevated serum BAs, particularly CDCA, in obese patients with relatively preserved endothelial function prompted investigation into the underlying mechanisms involving key BA receptors, TGR5 (*GPBAR1* encodes for TGR5) and FXR (*NR1H4* encodes for FXR). To explore the role of BAs and their receptors in endothelium-dependent vasodilation, we assessed arterioles from an additional 33 NHO patients (13 MHO, 20 MUO; baseline characteristics in Supplementary data online, *Table S3*) using *ex vivo* wire myograph (*Figure 3A*).

We firstly pre-treated MHO patient arterioles with the FXR agonist GW4064, the TGR5 agonist INT-777, and 6 representative BAs known to modulate FXR or TGR5 [FXR agonists: CDCA, TCDCA, GCDCA; TGR5 agonists: lithocholic acid (LCA), deoxycholic acid (DCA); FXR antagonist: tauroursodeoxycholic acid (TUDCA)] (*Figure 3B* and *E*). Endothelial dysfunction was evaluated using four metrics: AUC-ACH (*Figure 3C*), MAX-ACH (*Figure 3D*), AUC-BK (*Figure 3F*), and MAX-BK (*Figure 3G*). Notably, FXR agonists and FXR-activating BAs exerted relatively strong stimulatory effects on vasodilation. Interestingly, TCDCA, a taurine-conjugated CDCA derivative, demonstrated a more pronounced effect than CDCA and GCDCA. In contrast, TGR5 agonists (INT-777, LCA, DCA) and FXR antagonist TUDCA showed limited effects. Comparable findings were observed in MUO patient arterioles, where BA efficacy followed a similar trend (*Figure 3H–M*).

Furthermore, we tested the effects of different BAs, GW4064 and INT-777 on obesity-induced ED in diet-induced obese (DIO) mice. C57BL/6J male mice were fed with high-fat diet (HFD) for 12 weeks (see Supplementary data online, *Figure S8A*). Endothelial dysfunction was alleviated by GW4064 and TCDCA, whereas the INT-777, TUDCA, and tauro-β-muricholic acid (TβMCA, a rodent FXR antagonist) showed no effects on obesity-induced ED (see Supplementary data online, *Figure S8B*–*I*). We also performed targeted metabolomics on serum from normal chow diet mice, 4-week HFD mice, and 12-week HFD mice to quantify serum BA levels (see Supplementary data online, *Figure S9*). Our analysis revealed a general trend of decreased BAs, including CDCA, with prolonged HFD feeding in mice. This decrease in BAs in obese mice, particularly for CDCA which correlated with vasodilation in our human cohort, aligns with some existing literature suggesting reduced circulating BA pools in obese mice.^23^

Taken together, in *ex vivo* arteries from NHO patients or DIO mice, pre-treatment with BAs possessing FXR-activating properties ameliorated ED. Consistent with the results from metabolomics profile, CDCA displayed a robust capacity to alleviate obesity-induced ED. Notably, TCDCA emerged as the most effective BA.

### Taurochenodeoxycholic acid upregulates serine and one-carbon metabolism gene transcription in endothelial cells via FXR-ATF4 pathway

**Figure 4:**
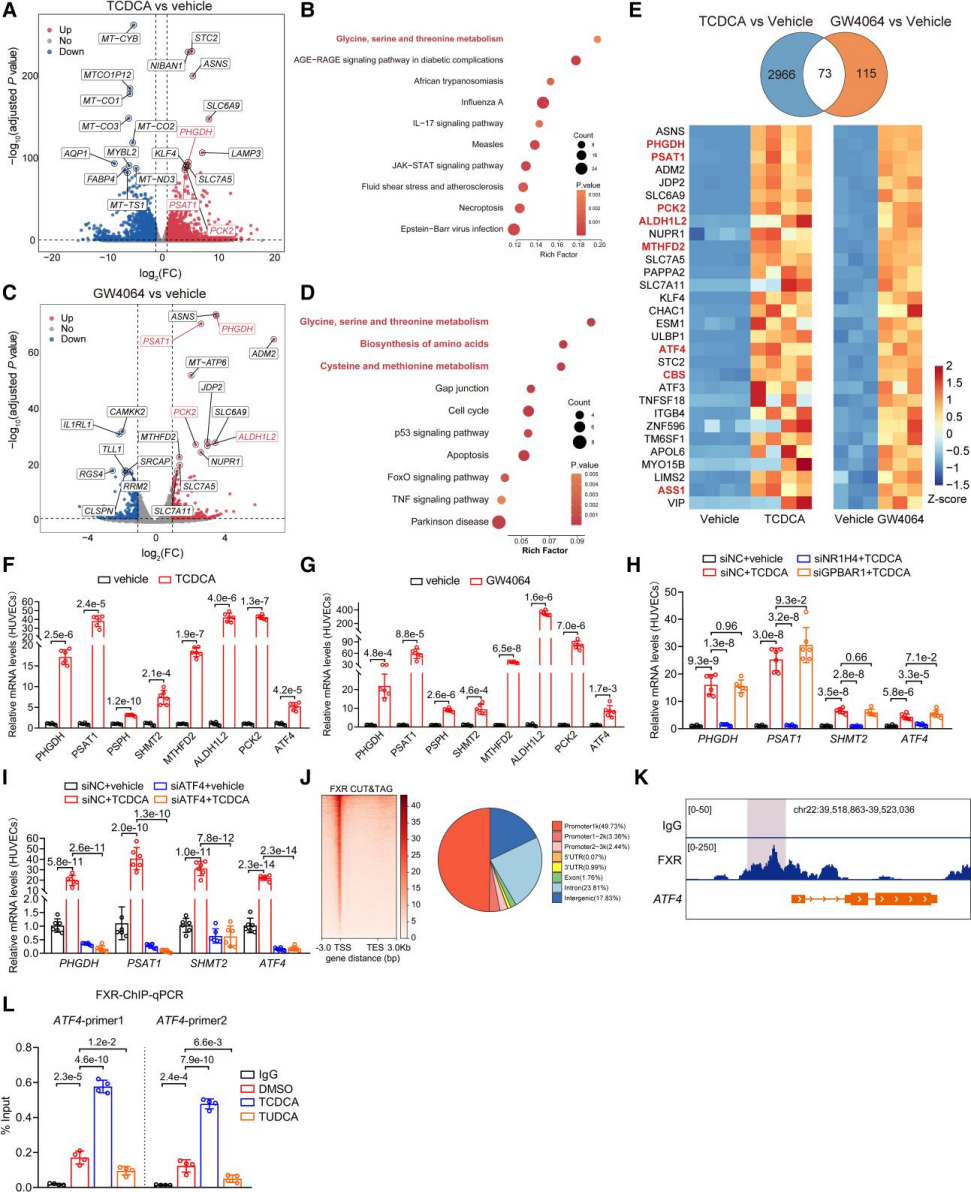
Taurochenodeoxycholic acid upregulates serine and one-carbon metabolism gene transcription in endothelial cells via FXR-ATF4 pathway. (*A*) Volcano plot displaying differentially expressed genes in human umbilical vein endothelial cells treated with taurochenodeoxycholic acid (50 μM, 24 h) vs vehicle control (*n* = 4). Genes meeting significance thresholds (adjusted *P* < .05 and |log₂ FC| ≥ 1) are highlighted in red (upregulated) and blue (downregulated). The top 20 most significant genes (lowest adjusted *P* values) are labelled with gene symbols. Dashed lines indicate significance cut-offs. (*B*) Bubble plot showing significantly enriched KEGG pathways among genes upregulated by taurochenodeoxycholic acid treatment (adjusted *P* < .05, log₂FC ≥ 1) in HUVECs. (*C*) Volcano plot displaying differentially expressed genes in human umbilical vein endothelial cells treated with GW4064 (10 μM, 24 h) vs vehicle control (*n* = 3). Genes meeting significance thresholds (adjusted *P* < .05 and |log₂ FC| ≥ 1) are highlighted in red (upregulated) and blue (downregulated). The top 20 most significant genes (lowest adjusted *P* values) are labelled with gene symbols. Dashed lines indicate significance cut-offs. (*D*) Bubble plot showing significantly enriched KEGG pathways among genes upregulated by GW4064 treatment (adjusted *P* < .05, log₂FC ≥ 1) in human umbilical vein endothelial cells. (*E*) Venn diagram showing the intersection of upregulated differentially expressed genes from the two RNA-seq data (upper). Heatmap (lower) of the top 30 most significantly upregulated genes (by adjusted *P* value) in the shared gene set. Rows represent genes, and columns represent biological replicates. (*F–G*) qPCR analysis of the mRNA levels of *PHGDH*, *PSAT1*, *PSPH*, *SHMT2*, *MTHFD2*, *ALDH1L2*, *PCK2*, and *ATF4* in human umbilical vein endothelial cells treated with taurochenodeoxycholic acid (50 μM, 24 h, *F*) or GW4064 (10 μM, 24 h, *G*) normalized to vehicle (*n* = 6). (*H*) siRNA experiments (siNR1H4 or siGPBAR1) followed by qPCR analysis of the mRNA levels of *PHGDH*, *PSAT1*, *SHMT2*, and *ATF4* in human umbilical vein endothelial cells normalized to siNC + vehicle (*n* = 6). (*I*) siRNA experiments (siATF4) followed by qPCR analysis of the mRNA levels of *PHGDH*, *PSAT1*, *SHMT2*, and *ATF4* in human umbilical vein endothelial cells normalized to siNC + vehicle (*n* = 6). (*J*) Heatmap showing normalized FXR-CUT&TAG intensity profiles across regions spanning 3 kb upstream of the transcription start site, through the gene body, to 3 kb downstream of the transcription end site for Flag-FXR-bound genes in human umbilical vein endothelial cells (overexpressed by Flag-tagged Farnesoid X receptor). Pie chart illustrating the genome-wide distribution of Farnesoid X receptor occupancy across promoter, exon, intron, and intergenic regions. (*K*) Representative Integrative Genomics Viewer (IGV) track displaying FXR-CUT&TAG signal and peak calls at the *ATF4* locus in human umbilical vein endothelial cells. (*L*) Chromatin immunoprecipitation qPCR showing the enrichment of Farnesoid X receptor on promoter regions of *ATF4* in human umbilical vein endothelial cells treated with vehicle, taurochenodeoxycholic acid (50 μM, 24 h), or tauroursodeoxycholic acid (50 μM, 24 h) (*n* = 4). Data are presented as mean ± standard deviation. Statistical analysis was performed using Student’s *t* test or Student’s *t* test with Welch correction (*F* and *G*), two-way ANOVA followed by Tukey’s *post hoc* test (*H* and *I*) and one-way ANOVA followed by Tukey’s multiple comparisons test (*L*)

**Figure 5:**
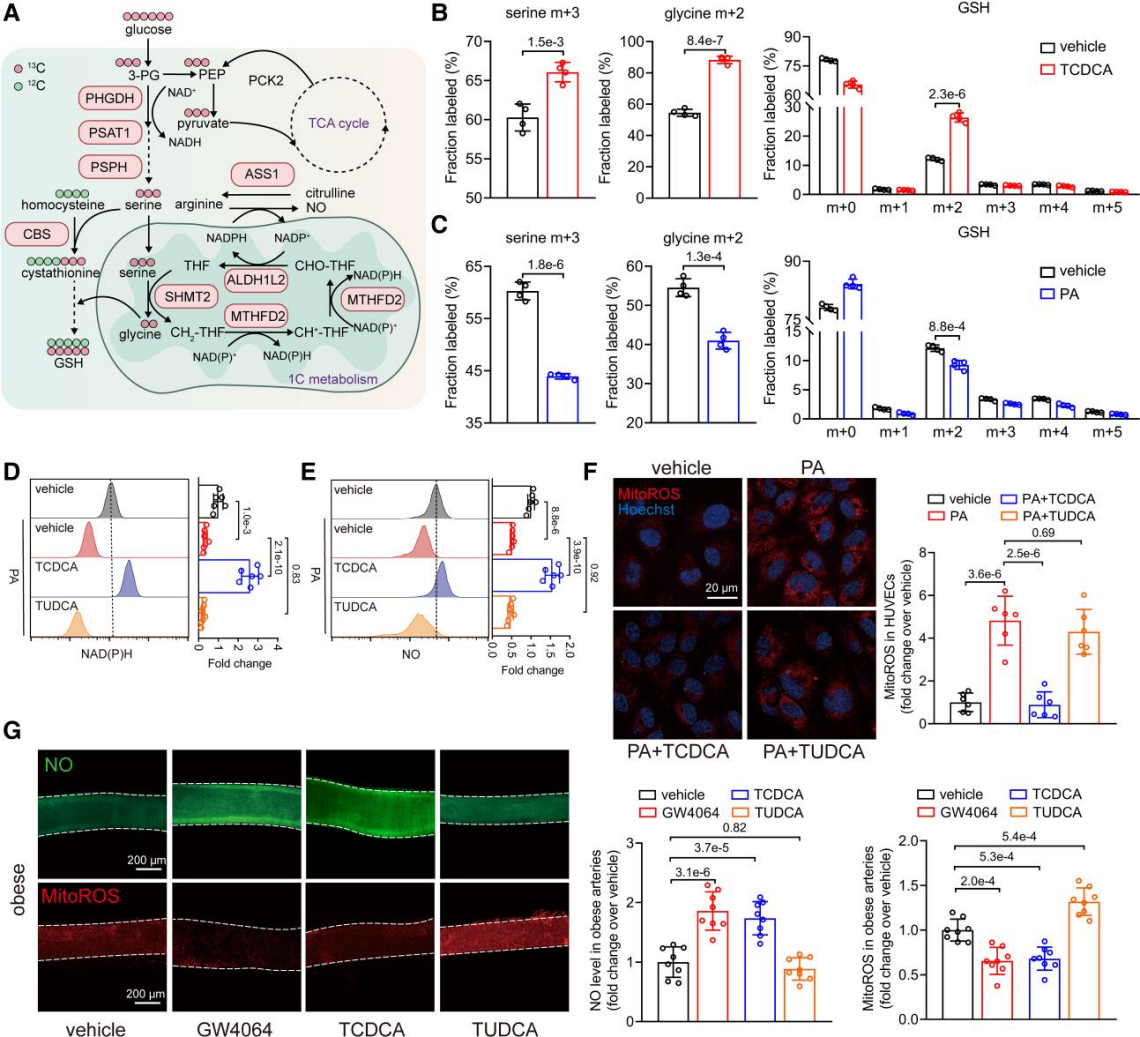
Taurochenodeoxycholic acid regulates redox homeostasis and nitric oxide release via driving serine and one-carbon metabolism. (*A*) Schematic diagram for serine, one-carbon metabolism and [U-^13^C]-glucose isotype tracing. (*B* and *C*) Fractional contribution of [U-^13^C] glucose carbon into the serine (m + 3), glycine (m + 2), and GSH (m + 0 to m + 5) in human umbilical vein endothelial cells treated with taurochenodeoxycholic acid (50 μM, 24 h, *B*) or PA (0.3 mM, 24 h, *C*) compared with vehicle (*n* = 4). (*D* and *E*) Representative flow cytometric images and analysis of NAD(*P*)H (*D*) and nitric oxide (*E*) levels in human umbilical vein endothelial cells treated with taurochenodeoxycholic acid (50 μM, 24 h), tauroursodeoxycholic acid (50 μM, 24 h), or palmitic acid (0.3 mM, 24 h) normalized to vehicle (*n* = 6). (*F*) Representative images of mitoROS staining in human umbilical vein endothelial cells treated with taurochenodeoxycholic acid (50 μM, 24 h), tauroursodeoxycholic acid (50 μM, 24 h), or palmitic acid (0.3 mM, 24 h) (scale bar = 20 µm), along with quantified mitoROS levels normalized to the vehicle (*n* = 6). (*G*) Representative images of mitoROS and nitric oxide staining in *ex vivo* human arterioles treated with taurochenodeoxycholic acid (50 μM), or tauroursodeoxycholic acid (50 μM) (scale bar = 200 µm), along with quantified mitoROS and NO levels normalized to the vehicle (*n* = 8). Data are presented as mean ± standard deviation. Statistical analysis was performed using Student’s *t* test (*B* and *C*), one-way ANOVA followed by Tukey’s multiple comparisons test (*G*), and two-way ANOVA followed by Tukey’s multiple comparisons test (*D–F*)

Regarding the role of TCDCA in alleviating obesity-induced ED, we sought to identify the key targets that could directly clarify the biological effects of TCDCA. We carried out RNA-seq of human umbilical vein ECs (HUVECs) treated with vehicle or TCDCA. A total of 5285 differentially expressed genes (DEGs) were observed (*Figure 4A*). We noticed that the ‘glycine, serine and threonine metabolism’ pathway was predominantly activated (*Figure 4B*). To further explore the role of TCDCA and identify its endothelial receptor, we performed RNA-seq on HUVECs treated with vehicle or GW4064 (*Figure 4C*). KEGG analysis showed that upregulated DEGs were enriched in amino acid metabolism pathways, including ‘glycine, serine and threonine metabolism’, ‘biosynthesis of amino acids’, and ‘cysteine and methionine metabolism’ (*Figure 4D*). Intersecting the upregulated DEGs from the two RNA-seq datasets identified 73 common genes (*Figure 4E*). Analysis of the top 30 common genes showed exclusive upregulation of genes involved in serine biosynthesis and one-carbon metabolism (*Figure 4E*, referred to *Figure 5A*). These findings underscore that TCDCA and FXR converge on these specific metabolic pathways in ECs. RNA-seq results were validated by qPCR (*Figure 4F* and *G*). Furthermore, siRNA-mediated knockdown confirmed that TCDCA regulates these key metabolic genes primarily via FXR, not TGR5 (*Figure 4H*).

The transcription factor ATF4 has been reported to regulate the expression of the key enzymes involved in serine metabolism (PHGDH, PSAT1, and SHMT2)^24^ and one-carbon metabolism (MTHFD2, ALDH1L2).^25^ Knockdown of ATF4 in HUVECs treated with TCDCA led to a significant downregulation in the mRNA levels of *PHGDH*, *PSAT1*, and *SHMT2* (*Figure 4I*). As a nuclear receptor and transcription factor, FXR can regulate gene expression upon TCDCA activation.^26^ To determine if FXR directly regulates *ATF4* transcription, we performed cleavage under targets and tagmentation (CUT&TAG) assay using Flag antibody in HUVECs overexpressing Flag-tagged FXR. Farnesoid X receptor was enriched in promoter regions (*Figure 4J*), and CUT&TAG tracks demonstrated specific FXR localization at the *ATF4* promoter (*Figure 4K*). Chromatin immunoprecipitation (ChIP) combined with qPCR analysis further validated that TCDCA treatment significantly enhanced the enrichment of FXR at the *ATF4* promoter. In contrast, treatment with TUDCA resulted in a reduction of FXR binding to the promoter (*Figure 4L*).

Further corroborating direct FXR regulation of ATF4, CUT&TAG using anti-FXR antibody in HUVECs treated with TCDCA or vehicle demonstrated that TCDCA significantly enhanced endogenous FXR enrichment at the *ATF4* promoter (see Supplementary data online, *Figure S10A* and *B*). We intersected genes upregulated by TCDCA in RNA-seq with genes bound by FXR in their promoter regions under TCDCA treatment, identifying 170 common genes (see Supplementary data online, *Figure S10C*). KEGG analysis of these common genes revealed a distinct set of pathways compared with the broader metabolic enrichment identified by TCDCA (*Figure 4B*) and GW4064 (*Figure 4D*) RNA-seq. This critical distinction underscores ATF4’s role as a central downstream effector linking FXR transcriptional targets to the observed broader metabolic shifts. We concluded that TCDCA targets serine metabolism and one-carbon metabolism via ATF-regulating transcription in ECs.

Acknowledging ATF4 as a marker of endoplasmic reticulum (ER) stress,^27^ we specifically investigated whether TCDCA induces ER stress in ECs and if ATF4 upregulation was secondary to this. Taurochenodeoxycholic acid treatment for 12 or 24 h significantly increased ATF4 protein levels without affecting other key ER stress markers (see Supplementary data online, *Figure S11A* and *B*). Co-treatment with ER stress inhibitors (4-PBA or GSK2606414) did not block TCDCA-induced increases in ATF4 protein or mRNA, or its downstream genes (see Supplementary data online, *Figure S11C*–*F*), confirming ATF4 upregulation is independent of ER stress. Taurochenodeoxycholic acid significantly reduced palmitic acid (PA)–induced ROS production (see Supplementary data online, *Figure S11G*). Investigation into the TCDCA-FXR-ATF4 axis role in endothelial quiescence showed TCDCA had minimal impact on EC proliferation (see Supplementary data online, *Figure S12A*) and migration (see Supplementary data online, *Figure S12B*).

### Taurochenodeoxycholic acid regulates redox homeostasis and nitric oxide release via driving serine and one-carbon metabolism

Serine, a non-essential amino acid synthesized *de novo* from glycolysis, is a crucial carbon source for one-carbon metabolism (*Figure 5A*).^28^ Our RNA-seq data suggested that TCDCA treatment enhances intracellular serine *de novo* synthesis, thereby driving one-carbon metabolism. We tested this hypothesis using [U-^13^C]-glucose stable isotope tracing combined with ultra-performance liquid chromatography-mass spectrometry (UPLC-MS). Taurochenodeoxycholic acid treatment significantly increased m + 3 serine and m + 2 glycine levels (*Figure 5B*), validating enhanced serine biosynthesis and flux into one-carbon metabolism. Serine biosynthesis and one-carbon metabolism are coupled with cysteine biosynthesis where the key enzyme CBS was upregulated in TCDCA-treated ECs (*Figure 4E*).^29^ This likely promotes the production of glutathione (GSH), a critical cellular antioxidant synthesized from three amino acids (glutamate, cysteine, and glycine).^30^ Our data demonstrated that TCDCA markedly elevated total m + 2 GSH levels (*Figure 5B*). We also observed that PA, an obesity-related saturated fatty acid related with obesity,^31^ reduced the intracellular levels of *de novo* synthesized serine and glycine and m + 2 GSH (*Figure 5C*).

Based on RNA-seq data and metabolic pathway analysis, we identified several key enzymes that mediates NAD(P)H generation including PHGDH, MTHFD2, and ALDH1L2 (*Figure 5A*). Our data further demonstrated that TCDCA, but not TUDCA, elevated NAD(P)H level in PA-treated ECs (*Figure 5D*). Moreover, mitochondrial reactive oxygen species (mitoROS) levels, which were elevated by PA, were notably reduced by TCDCA treatment, whereas TUDCA had no such effect (*Figure 5F*). We observed that serine and one-carbon metabolism fed NAD(P)H and GSH generation, contributing to the reduction of mitoROS. Diminished nitric oxide (NO) bioavailability plays a critical role in the development of ED and hypertension.^32^ Depleted NAD(P)H levels and an imbalance in ROS can directly impair the NO production.^33^ Flow cytometry analysis revealed that TCDCA treatment elevated NO levels in PA-treated ECs (*Figure 5E*). To further validate the role of FXR, TGR5, and ATF4 in ECs, siRNA experiments indicated that silencing FXR and ATF4 inhibited the effects of TCDCA, whereas knockdown of TGR5 had no effect (see Supplementary data online, *Figure S13*). Consistent with the *in vitro* experiments, production of mitoROS and NO in *ex vivo* NHO patient arterioles confirmed TCDCA and GW4064 regulates redox homeostasis and NO release (*Figure 5G*). Meanwhile, detection of mitoROS and NO on DIO mice mesenteric arteries demonstrated the effects of BAs on mice endothelium-dependent vasodilation (see Supplementary data online, *Figure S8J*). The above results suggest that TCDCA regulates endothelial redox homeostasis and NO release by driving serine and one-carbon metabolism.

### PHB1 is a novel binding protein of Farnesoid X receptor and inhibits *ATF4* transcription

**Figure 6:**
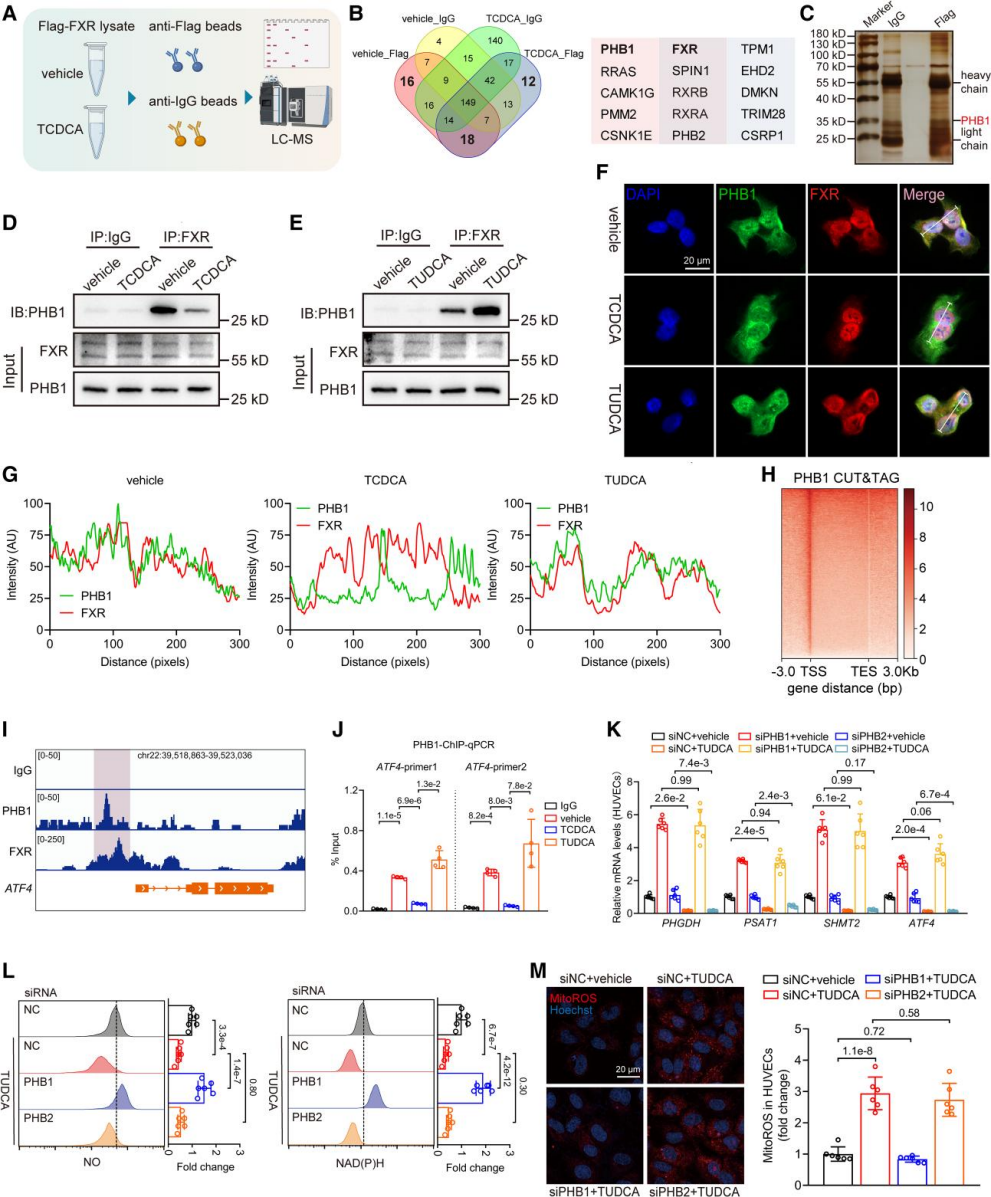
PHB1 is a novel binding protein of Farnesoid X receptor and inhibits *ATF4* transcription. (*A*) Flow chart detailing the experimental protocol for immunoprecipitation from human umbilical vein endothelial cells transduced with adenovirus expressing Flag-FXR. The protocol involves subsequent treatment with vehicle or taurochenodeoxycholic acid (50 μM, 24 h), immunoprecipitation using anti-Flag or anti-IgG magnetic beads, and analysis by liquid chromatography-mass spectrometry. (*B*) Venn diagram displaying the overlap among four groups and table listing the top 5 ranked proteins from the three highlighted overlapping regions. (*C*) Lysates from human umbilical vein endothelial cells transduced with adenovirus expressing Flag-FXR were purified with anti-Flag or anti-IgG magnetic beads and silver-stained after resolving by SDS-PAGE. (*D* and *E*) Immunoprecipitation and western blots showing the interaction between PHB1 and Farnesoid X receptor in human umbilical vein endothelial cells treated with vehicle, taurochenodeoxycholic acid (50 μM, 24 h, *D*), or tauroursodeoxycholic acid (50 μM, 24 h, *E*) compared with vehicle. (*F*) Representative immunofluorescence images for detecting nucleus (DAPI), PHB1, and FXR in human umbilical vein endothelial cells treated with vehicle, taurochenodeoxycholic acid (50 μM, 24 h), or tauroursodeoxycholic acid (50 μM, 24 h) (scale bar = 20 µm). (*G*) Analysis of the colocalization of Farnesoid X receptor and PHB1 in human umbilical vein endothelial cells treated with vehicle, taurochenodeoxycholic acid (50 μM, 24 h), or tauroursodeoxycholic acid (50 μM, 24 h). (*H*) Heatmap showing normalized PHB1 CUT&TAG intensity profiles across regions spanning 3 kb upstream of the transcription start site, through the gene body, to 3 kb downstream of the transcription end site for PHB1-bound genes in human umbilical vein endothelial cells. (*I*) Integrative Genomics Viewer analysis representing IgG, PHB1, and Farnesoid X receptor peaks at the *ATF4* loci in human umbilical vein endothelial cells. (*J*) Chromatin immunoprecipitation qPCR showing the enrichment of PHB1 on promoter regions of *ATF4* in human umbilical vein endothelial cells treated with vehicle, taurochenodeoxycholic acid (50 μM, 24 h), or tauroursodeoxycholic acid (50 μM, 24 h) (*n* = 4). (*K*) siRNA experiments (siPHB1 or siPHB2) followed by qPCR analysis of the mRNA levels of *PHGDH*, *PSAT1*, *SHMT2*, and *ATF4* in human umbilical vein endothelial cells treated with vehicle or tauroursodeoxycholic acid (50 μM, 24 h) normalized to siNC + vehicle (*n* = 6). (*L*) Representative flow cytometry analysis showing NAD(P)H and nitric oxide levels in human umbilical vein endothelial cells following siRNA-mediated knockdown of PHB1 or PHB2 and treatment with vehicle or tauroursodeoxycholic acid (50 μM, 24 h), normalized to siNC (*n* = 6). (*M*) Representative images of mitoROS staining in human umbilical vein endothelial cells following siRNA-mediated knockdown of PHB1 or PHB2 and treatment with vehicle or tauroursodeoxycholic acid (50 μM, 24 h) (scale bar = 20 µm), along with quantified mitoROS levels normalized to siNC + vehicle (*n* = 6). Data are presented as mean ± standard deviation. Statistical analysis was performed using the Brown-Forsythe ANOVA test followed by Dunnett’s T3 multiple comparisons test (*J*) and two-way ANOVA followed by Tukey’s multiple comparisons test (*K–M*)

To elucidate the mechanism by which TCDCA-activated FXR regulates *ATF4* transcription, we screened for potential FXR-interacting proteins. Human umbilical vein endothelial cells overexpressing Flag-tagged FXR were treated with TCDCA or vehicle, and cell lysates were subjected to anti-Flag or anti-IgG immunoprecipitation (IP) followed by LC-MS (*Figure 6A*). We exclusively identified 16 proteins in vehicle-treated cells that were absent in TCDCA-treated cells, suggesting their interaction with inactive FXR. Prohibitin-1 (PHB1) ranked highest among these potential FXR-interacting proteins (*Figure 6B* and *C*). PHB1 is known to localize to the nucleus and modulate transcription factor activity.^34^ Immunoprecipitation and western blot analysis further validated the interaction between endogenous PHB1 and FXR in HUVECs. Consistent with the LC-MS screen, TCDCA treatment markedly reduced the interaction between PHB1 and FXR compared with vehicle control (*Figure 6D*). Conversely, treatment with TUDCA enhanced the interaction (*Figure 6E*). The subcellular distribution of FXR and PHB1 was confirmed using IF staining, showing nuclear colocalization of FXR and PHB1 in ECs under normal conditions (*Figure 6F* and *G*). Taurochenodeoxycholic acid treatment resulted in reduced nuclear enrichment of PHB1, while TUDCA maintained strong nuclear colocalization of FXR and PHB1.

To further evaluate the role of PHB1, we performed CUT&TAG assay to evaluate the DNA-binding profiles of PHB1 in ECs (*Figure 6H*). Representative snapshots of CUT&TAG tracks showed the colocalization of FXR and PHB1 at *ATF4* promoter in ECs, suggesting a potential collaborative regulatory role of FXR and PHB1 in modulating downstream target genes (*Figure 6I*). ChIP-qPCR analyses confirmed the binding of PHB1 to the promoter regions of *ATF4*. As expected, the enrichment was reduced in TCDCA-treated ECs, whereas TUDCA treatment enhanced the binding of PHB1 to the promoter (*Figure 6J*). To further determine whether PHB1 regulates the transcription of *ATF4* and its downstream serine metabolism genes, we utilized siRNA to knockdown PHB1 or its family member PHB2. Notably, the mRNA levels of *PHGDH*, *PSAT1*, *SHMT2*, and *ATF4* were decreased by TUDCA; however, this reduction was abolished upon knockdown of PHB1, but not PHB2 (*Figure 6K*). These results suggest that PHB1 in the nucleus binds to FXR, suppressing the transcription of *ATF4* and its downstream genes. Taurochenodeoxycholic acid disrupts this interaction, thereby enhancing the transcription of downstream genes.

To further investigate the role of PHB1 in obesity-induced ED, we performed IF staining of PHB1, along with FXR, ATF4, and TGR5, in human arterioles from NHO patients stratified into Q1 and Q4 groups. Quantitative analysis demonstrated both FXR (see Supplementary data online, *Figure S14A*) and ATF4 (see Supplementary data online, *Figure S14C*) maintained stable expression levels in CD31^+^ ECs between Q1 and Q4 groups. TGR5 showed negligible expression in CD31^+^ ECs (see Supplementary data online, *Figure S14B*). In contrast, PHB1 was upregulated in the Q4 group (see Supplementary data online, *Figure S14D*). Consistent with this finding, elevated PHB1 expression was also observed in the aortic endothelium of HFD mice (see Supplementary data online, *Figure S14E*). *Ex vivo* PHB1 knockdown in mesenteric arteries from DIO mice resulted in a modest but statistically significant improvement in endothelium-dependent vasodilation (see Supplementary data online, *Figure S15*), providing support for PHB1’s regulatory role in obesity-induced ED.

### Endothelial Farnesoid X receptor critically mediates vertical sleeve gastrectomy–induced alleviation of obesity-induced endothelial cell

**Figure 7:**
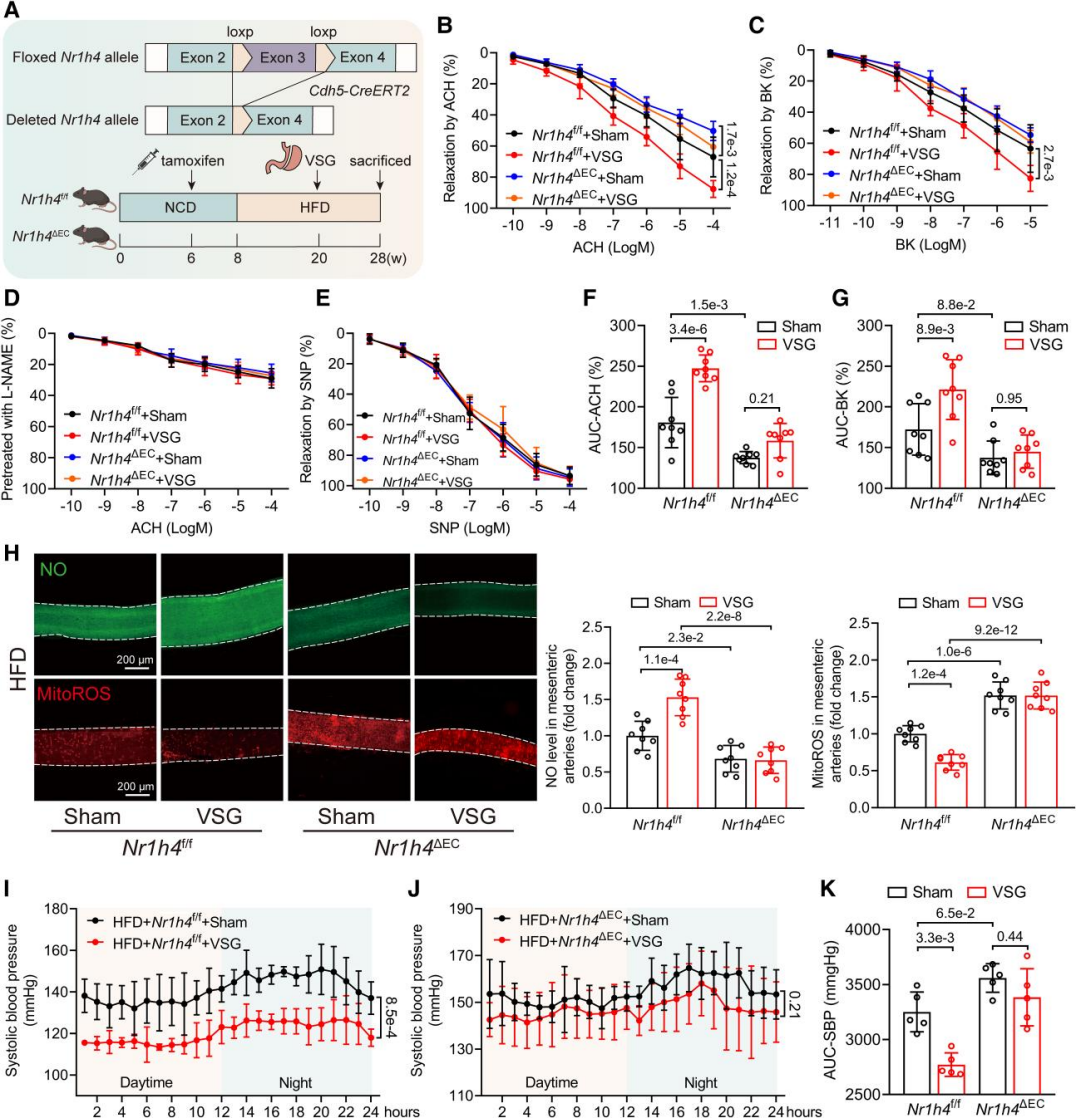
Endothelial Farnesoid X receptor critically mediates vertical sleeve gastrectomy–induced alleviation of obesity-induced endothelial dysfunction. (*A*) Schematic diagram of transgenic mice used to generate endothelial-specific Farnesoid X receptor knockout (*Nr1h4*^ΔEC^) mice and sham- or vertical sleeve gastrectomy–treated mice experiment flow chart. (*B–C*) Endothelium-dependent vasodilation to cumulative concentration of acetylcholine (*B*) or bradykinin (*C*) in sham- or vertical sleeve gastrectomy–treated *Nr1h4*^f/f^ or *Nr1h4*^ΔEC^ mice mesenteric arteries (*n* = 8). (*D–E*) Endothelium-independent vasodilation to cumulative concentration of acetylcholine pre-treated with L-NAME (*D*) or to cumulative concentration of sodium nitroprusside (*E*) in sham- or vertical sleeve gastrectomy–treated *Nr1h4*^f/f^ or *Nr1h4*^ΔEC^ mice mesenteric arteries (*n* = 8). (*F–G*) AUC-ACH (*F*) and AUC-BK (*G*) levels in sham- or vertical sleeve gastrectomy–treated *Nr1h4*^f/f^ or *Nr1h4*^ΔEC^ mice mesenteric arteries (*n* = 8). (*H*) Representative images of mitoROS and nitric oxide staining in sham- or vertical sleeve gastrectomy–treated *Nr1h4*^f/f^ or *Nr1h4*^ΔEC^ mice mesenteric arteries (scale bar = 200 µm), along with quantified mitoROS and nitric oxide levels normalized to the *Nr1h4*^f/f^ + sham group (*n* = 8). (*I*) Systolic blood pressure levels monitored by 24 h telemetry measurements in 28-week-old *Nr1h4*^f/f^ mice (sham vs vertical sleeve gastrectomy, *n* = 5). (*J*) Systolic blood pressure levels monitored by 24 h telemetry measurements in 28-week-old *Nr1h4*^ΔEC^ mice (sham vs vertical sleeve gastrectomy, *n* = 5). (*K*) AUC-SBP in sham- or vertical sleeve gastrectomy–treated *Nr1h4*^f/f^ or *Nr1h4*^ΔEC^ mice (*n* = 5). Data are presented as mean ± standard deviation. Statistical analysis was performed using two-way ANOVA followed by Tukey’s multiple comparisons test (*B*, *C*, *F*, *G*, *H*, and *K*) and repeated measures two-way ANOVA followed by Bonferroni multiple comparisons test (*I* and *J*)

Bariatric surgery can reverse obesity-induced ED^35^ and serve as an effective strategy for BP control in patients with both obesity and hypertension.^36^ Previous studies highlight BAs and FXR signalling as key molecular mechanisms underlying the beneficial effects of bariatric surgery.^37^ Given the central role of FXR in obesity-induced ED in this study, we hypothesized that endothelial FXR mediates the beneficial effects of bariatric surgery on ED and hypertension. To test this, we performed VSG on 12-week HFD *Nr1h4*^f/f^ or *Nr1h4*^ΔEC^ mice and then assessed vasodilation in the mesenteric arteries after 8 weeks (*Figure 7A*). Endothelium-dependent relaxation in response to ACH (*Figure 7B*) and BK (*Figure 7C*) demonstrated that VSG ameliorated obesity-induced ED and EC-specific FXR deficiency exacerbated ED and abolished the beneficial effects of VSG. Endothelium-independent vasodilation showed no differences among the four groups (*Figure 7D* and *E*). AUC-ACH (*Figure 7F*) and AUC-BK (*Figure 7G*) further confirmed the above findings. The ED observed across the four groups was associated with changes in mitoROS and NO levels of *ex vivo* mesenteric arteries (*Figure 7H*). Consistent with ED, telemetric monitoring of 24 h BP revealed that VSG alleviated obesity-induced hypertension in *Nr1h4*^f/f^ mice (*Figure 7I–K*). Conversely, EC-specific FXR deficiency exacerbated baseline hypertension and impaired the antihypertensive effects of VSG.

To further determine the effects of endothelial FXR, we developed adeno-associated viral vector serotype 9 (AAV9) expressing ICAM2 promoter-driven *Nr1h4* for EC-specific FXR overexpression in HFD mice (see Supplementary data online, *Figure S16A*). High-fat diet mice injected with AAV9-Ctrl or AAV9-*Nr1h4* were subjected to sham or VSG surgery. We observed that EC-specific FXR overexpression or VSG alleviated ED (see Supplementary data online, *Figure S16B*–*E*). Furthermore, AUC-ACH (see Supplementary data online, *Figure S16F*) and AUC-BK (see Supplementary data online, *Figure S16G*) demonstrated an improvement of ED in AAV9-*Nr1h4* + VSG group compared with AAV9-*Nr1h4* +sham or AAV9-Ctrl + VSG groups. Consistently, mitoSOX and NO staining in mesenteric arteries revealed that EC-specific FXR overexpression markedly restored endothelial redox homeostasis and promoted NO release (see Supplementary data online, *Figure S16H*).

Collectively, these findings strongly underscore the critical role of endothelial FXR as an intrinsic protective factor against obesity-induced ED and hypertension and highlight its ability to amplify the therapeutic benefits of bariatric surgery.

### Taurochenodeoxycholic acid alleviates obesity-induced endothelial dysfunction and hypertension by activating endothelial Farnesoid X receptor

**Figure 8:**
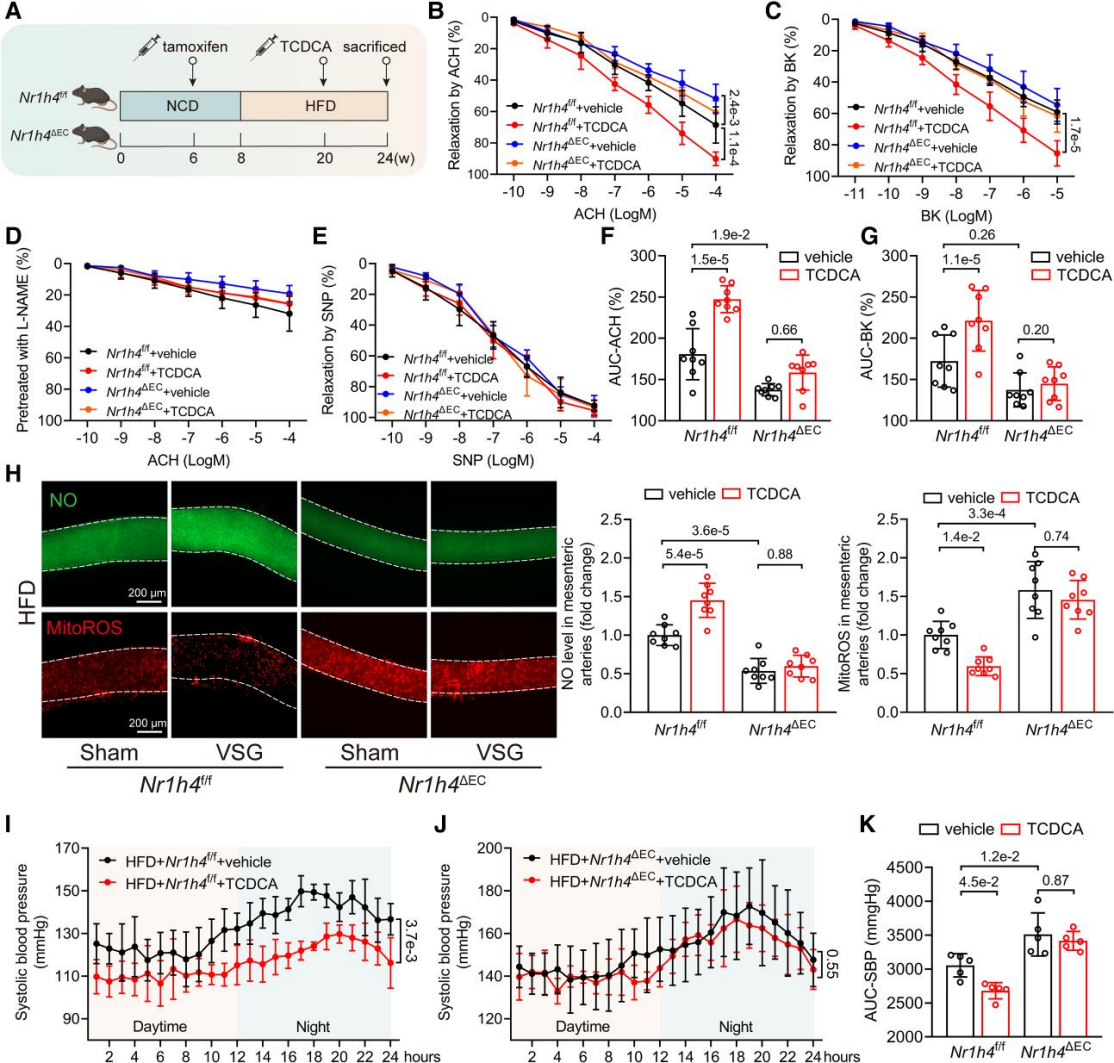
Taurochenodeoxycholic acid alleviates obesity-induced endothelial dysfunction and hypertension by activating endothelial Farnesoid X receptor. (*A*) Vehicle- or taurochenodeoxycholic acid–treated *Nr1h4*^f/f^ or *Nr1h4*^ΔEC^ mice experiment flow chart. (*B–C*) Endothelium-dependent vasodilation to cumulative concentration of acetylcholine (*B*) or bradykinin (*C*) in vehicle- or taurochenodeoxycholic acid–treated *Nr1h4*^f/f^ or *Nr1h4*^ΔEC^ mice mesenteric arteries (*n* = 8). (*D–E*) Endothelium-independent vasodilation to cumulative concentration of acetylcholine pre-treated with L-NAME (*D*) or to cumulative concentration of sodium nitroprusside (*E*) in vehicle- or taurochenodeoxycholic acid–treated *Nr1h4*^f/f^ or *Nr1h4*^ΔEC^ mice mesenteric arteries (*n* = 8). (*F–G*) AUC-ACH (*F*) and AUC-BK (*G*) levels in vehicle- or taurochenodeoxycholic acid–treated *Nr1h4*^f/f^ or *Nr1h4*^ΔEC^ mice mesenteric arteries (*n* = 8). (*H*) Representative images of mitoROS and nitric oxide staining in vehicle- or taurochenodeoxycholic acid–treated *Nr1h4*^f/f^ or *Nr1h4*^ΔEC^ mice mesenteric arteries (scale bar = 200 µm), along with quantified mitoROS and nitric oxide levels normalized to the *Nr1h4*^f/f^ + vehicle group (*n* = 8). (*I*) Systolic blood pressure levels monitored by 24 h telemetry measurements in 24-week-old *Nr1h4*^f/f^ mice (vehicle vs taurochenodeoxycholic acid, *n* = 5). (*J*) Systolic blood pressure levels monitored by 24 h telemetry measurements in 24-week-old *Nr1h4*^ΔEC^ mice (vehicle vs taurochenodeoxycholic acid, *n* = 5). (*K*) AUC-SBP levels in vehicle- or TCDCA-treated diet-induced obese *Nr1h4*^f/f^ or *Nr1h4*^ΔEC^ mice (*n* = 5). Data are presented as mean ± standard deviation. Statistical analysis was performed using two-way ANOVA followed by Tukey’s multiple comparisons test (*B*, *C*, *F*, *G*, *H*, and *K*) and repeated measures two-way ANOVA followed by Bonferroni multiple comparisons test (*I* and *J*)

Given that FXR activation by TCDCA alleviated *ex vivo* obesity-induced ED, we proceeded to investigate its effects in *in vivo* obesity models. Twelve-week HFD mice were administered either vehicle or TCDCA under HFD treatment for an additional 4 weeks (*Figure 8A*). Endothelium-dependent vasodilation in response to ACH (*Figure 8B*) and BK (*Figure 8C*) revealed that TCDCA effectively alleviated ED, whereas EC-specific FXR deficiency negated these beneficial effects. Endothelium-independent vasodilation showed no significant differences among the four groups (*Figure 8D* and *E*). AUC-ACH (*Figure 8F*) and AUC-BK (*Figure 8G*) provided a clearer demonstration of the therapeutic effects of TCDCA and the role of endothelial FXR. *In vivo* administration of TCDCA reduced mitoROS levels and increased NO production in mesenteric arteries (*Figure 8H*). We performed telemetric monitoring of 24 h BP in HFD and TCDCA-treated HFD mice. Consistently, TCDCA administration decreased the SBP in obesity-induced hypertension mice (*Figures 8I* and *K*). In contrast, no statistical differences in SBP were observed between HFD + *Nr1h4*^ΔEC^ + vehicle and HFD + *Nr1h4*^ΔEC^ + TCDCA mice (*Figure 8J* and *K*), indicating the role of endothelial FXR.

Our findings suggest the potential of TCDCA for treating ED and hypertension in DIO mice. Given the substantial heterogeneity in endothelium-dependent vasodilation across different types of obesity and even within the same obesity subtype such as MHO and MUO, we tested the effects of TCDCA in other obese or diabetic mouse models: the *ob/ob* model (see Supplementary data online, *Figure S17*) and the *db/db* (see Supplementary data online, *Figure S18*) model.^38^ In each mouse model, we treated 8-week mice with TCDCA or vehicle for 4 weeks. Our data showed that TCDCA administration effectively mitigated obesity-induced ED and hypertension in either the *ob/ob* model or the *db/db* model. Notably, TCDCA treatment had no significant impact on body weight or glucose tolerance in these models. Echocardiography results showed that TCDCA alleviated cardiac dysfunction of *ob/ob* but not *db/db*, which was characterized by increasing ejection fraction, fractional shortening. Masson and periodic acid Schiff (PAS) staining were performed to assess TCDCA’s effects on cardiac and renal remodelling, with results indicating only a limited therapeutic benefit. To test the effects of TCDCA on atherosclerosis, TCDCA was administered to *ApoE*^−/−^ mice (see Supplementary data online, *Figure S19A*). The proportions of atherosclerotic surface lesions were reduced in the TCDCA-treated mice (see Supplementary data online, *Figure S19B*). Haematoxylin and eosin staining (HE) and oil red O (ORO)–stained aortic roots displayed decreased lesion areas in the TCDCA-treated mice (see Supplementary data online, *Figure S19C*).

In summary, our results demonstrate that TCDCA effectively protects mice against obesity-induced ED, hypertension and even atherosclerosis in different models, underscoring its broad protective effects in obesity.

## Discussion

Obesity disrupts vascular homeostasis, fostering pathological hypertension and increasing CVD risk and mortality.^39^ Endothelial cells play a central role in these obesity-induced metabolic and vascular changes due to their unique interface with circulating factors.^40^ While most studies assess obesity-induced ED using animal models or non-invasive human methods, wire myograph on *ex vivo* human arterioles remains the gold standard for evaluating vascular function, offering a unique bridge from laboratory research to clinical insights.^41^ Acknowledging valuable insights from a recent pivotal study on *ex vivo* human vessels investigating age- and obesity-induced ED, that work was limited by a small cohort (47 patients) and exclusive focus on a single protein (SIRT1).^8^ To address these limitations and identify key factors contributing to obesity-induced ED, we recruited a larger cohort of 213 patients with non-hypertensive obesity, specifically aiming to investigate ED at its earliest stages before the confounding influence of established hypertension.

Although obesity is generally linked to metabolic dysfunction and cardiometabolic diseases, certain individuals with obesity are protected from many adverse metabolic effects associated with excess body fat and are classified as MHO.^18^ In this study, MHO was defined as obesity with no more than one of the components of metabolic syndrome (excluding increased waist circumference): high BP, high FBG, high TG, and low HDL-C; all other NHO individuals were categorized as MUO. Consistent with existing literature, MUO patients exhibited more severe ED than MHO patients (*Figure 1B–F*). However, we note the observed difference in ED severity between MHO and MUO was relatively modest, underscoring that the metabolically healthy state may be transient and emphasizing the need for careful monitoring of vascular health even in MHO individuals. Despite considerable inter-individual heterogeneity in vasodilation within the NHO population, obesity-induced ED showed no significant correlation with many traditional cardiovascular risk factors. This lack of prediction by conventional measures prompted targeted serum metabolomic profiling, which identified BAs, especially CDCA, as potentially critical regulators of obesity-induced ED. Discovering reliable biomarkers capable of predicting early-stage obesity-induced ED is crucial for developing effective prevention and therapeutic approaches.

The effects of BAs and their receptors on vascular phenotypes are increasingly recognized, though debate persists regarding their precise roles in modulating vasodilation and impacting BP.^42^ Mechanistically, FXR is robustly expressed in ECs and its activation inhibits ET-1 release and partially regulates eNOS.^12,13,43^  *In vivo*, TCDCA and taurodeoxycholic acid infusions increase mesenteric arterial blood flow and induce systemic arterial hypotension, whereas TUDCA has no significant effect.^44^ Systemic FXR-deficient mice exhibit enhanced hypotension and vasodilation induced by ACH.^43^ However, these studies often focus on short-term vasodilatory effects, potentially neglecting long-term impacts, and systemic knockout models present confounding factors. In our study, we evaluated different BAs’ effects on vasodilation using *ex vivo* NHO human arterioles and mesenteric arteries from DIO mice pre-treated with BAs, GW4064 or INT-777. Taurochenodeoxycholic acid emerged as the most effective BA, demonstrating significant potential as a natural compound for alleviating obesity-induced ED. Furthermore, in knockout models, endothelial-specific FXR deletion exacerbated obesity-induced ED and hypertension, impaired physiological EC functions under obesity, and completely negated the beneficial effects of both VSG and TCDCA, underscoring the critical, protective role of endothelial FXR.

Serine and its metabolic pathways are fundamental to cellular survival and function, providing essential one-carbon units for biosynthesis and modifications.^45^ Recent studies underscore the pivotal role of endothelial serine metabolism in EC function, vascular homeostasis, and sprouting angiogenesis. For instance, endothelial-specific knockout of *Phgdh*, the committed enzyme in *de novo* serine biosynthesis, leads to depletion of heme, GSH, and NAD(P)H, impairing mitochondrial function.^46^ Serine metabolism is also crucial for perturbed endothelial homeostasis and aberrant vascularization in cancer,^47^ its biosynthesis prevents EC senescence,^48^ and signalling is linked to the pathogenesis of heart failure and dilated cardiomyopathy.^25,49^ In line with these findings, our study elucidated the critical role of serine metabolism and subsequent one-carbon metabolism in endothelial vasodilation and obesity-induced ED. RNA-seq and [U-^13^C]-glucose isotype tracing data showed that TCDCA-induced FXR activation significantly enhances these metabolic pathways by increasing key enzymes transcription. Our integrated RNA-seq and CUT&TAG analyses identified ATF4 as a key downstream transcription factor of FXR, regulating the expression of multiple enzymes within serine and one-carbon metabolism. Beyond its mitochondrial localization, PHB1 is also present in the nucleus, where it functions as a transcriptional regulator by interacting with transcription factors.^50,51^ Notably, we discovered that nuclear PHB1 directly binds to FXR, acting as a co-transcription factor to suppress the transcription of *ATF4* and its downstream target genes. Intriguingly, TCDCA binding to FXR disrupts this inhibitory PHB1-FXR interaction and reduces PHB1 nuclear localization, thereby relieving transcriptional repression and promoting *ATF4* and metabolic gene expression. Our study reveals an integrated role of the TCDCA-PHB1-FXR-ATF4 axis in regulating serine and one-carbon metabolism, which supports the maintenance of NAD(P)H, GSH, and ROS balance. This regulation is essential for driving NO synthesis, thereby mitigating the development of obesity-induced ED and hypertension.

Our study has several limitations that should be considered. Firstly, the findings regarding CDCA as a biomarker and preventive measure for obesity-induced ED in NHO patients are based on a single cohort. Therefore, the generalizability and robustness of these results require validation in independent cohorts with diverse demographic characteristics. Secondly, while we utilized the wire myograph to assess ED, future studies should incorporate a broader range of objective measures, such as flow-mediated dilation, to provide a more comprehensive evaluation of ED. The lack of multi-faceted assessment in our current study limits the scope of our conclusions.

## Supplementary Material

ehaf766_Supplementary_Data

## Declarations

### Data Availability

Bulk-RNA and CUT&TAG sequencing data were deposited in the Gene Expression Omnibus (GEO) database. Metabolic profiling of serum samples from patients cannot be shared publicly due to the privacy of individuals that participated in the study. The data will be shared on reasonable request to the corresponding author.

### Pre-registered Clinical Trial Number

None supplied.
