# Supplementary material for: Taurochenodeoxycholic acid alleviates obesity-induced endothelial dysfunction
Source: Eur Heart J. 2025 Oct 3;47(10):1221–38. doi: 10.1093/eurheartj/ehaf766 (PMC13017434; doi:10.1093/eurheartj/ehaf766)
Supplement: ehaf766_Supplementary_Data [file ehaf766_supplementary_data.pdf]

Detailed methods

Reagents and resources

Reagents or resources	Source	Identifier
Antibodies		
Mouse monoclonal anti-FLAG	Sigma-Aldrich	Cat#F1804, RRID: AB_262044
Mouse monoclonal anti-PHB1	Invitrogen	Cat#MA5-12858, RRID: AB_10980219
Mouse monoclonal anti-FXR	Invitrogen	Cat#417200, RRID: AB_2532196
Mouse monoclonal anti-p-eIF2α (Ser51)	Proteintech	Cat#68023-1-Ig, RRID: AB_2918767
Normal mouse IgG	Sigma-Aldrich	Cat#12-371, RRID: AB_145840
Rabbit polyclonal anti-CD31	Abcam	Cat#ab28364, RRID: AB_726362
Rabbit monoclonal anti-VCAM1	Abcam	Cat#ab134047, RRID: AB_2721053
Rabbit polyclonal anti-ATF4	Proteintech	Cat#10835-1-AP, RRID: AB_2058600
Rabbit polyclonal anti-Endothelin-1	Proteintech	Cat#12191-1-AP, RRID: AB_889392

Rabbit anti-p-PERK (Thr982)	Proteintech	Cat#82534-1-RR, RRID: AB_3085359
Rabbit polyclonal anti-PERK	Proteintech	Cat#20582-1-AP, RRID: AB_10695760
Rabbit polyclonal anti-eIF2α	Proteintech	Cat#11170-1-AP, RRID: AB_2096489
Rabbit polyclonal anti-CHOP	Proteintech	Cat#15204-1-AP, RRID: AB_2292610
Rabbit polyclonal anti-GAPDH	Proteintech	Cat#10494-1-AP, RRID: AB_2263076
Rabbit polyclonal anti-TGR5	Invitrogen	Cat#PA5-34262, RRID: AB_2551614
Sheep polyclonal anti-Von Willebrand Factor	Abcam	Cat#ab11713, RRID: AB_298501
Goat polyclonal anti-CD31		Cat#ab11713, RRID: AB_298501
Virus		
AAV9-*Nr1h4*	GeneChem	N/A
Ad-Flag-*NR1H4*	OBiO Technology	N/A
Chemicals, Peptides, and Recombinant proteins		
Taurochenodeoxycholic acid	Sigma-Aldrich	Cat#T6260, CAS:

		6009-98-9
Glycochenodeoxycholic acid	Sigma-Aldrich	Cat#50534; CAS: 16564-43-5
Chenodeoxycholic acid	Sigma-Aldrich	Cat#C9377; CAS: 474-25-9
Tauroursodeoxycholic acid	MedChemExpress	Cat#HY-19696; CAS: 14605-22-2
Lithocholic acid	Sigma-Aldrich	Cat#L6250; CAS: 434-13-9
Deoxycholic acid	Sigma-Aldrich	Cat#30960; CAS: 83-44-3
Tauro-β-muricholic acid	MedChemExpress	Cat#HY-135103; CAS: 145022-92-0
GW4064	MedChemExpress	Cat#HY-50108; CAS: 278779-30-9
INT-777	MedChemExpress	Cat#HY-15677; CAS: 1199796-29-6
Palmitic acid	MedChemExpress	Cat#HY-N0830; CAS: 57-10-3
Acetylcholine	Sigma-Aldrich	Cat#A6625; CAS: 60-31-1
Sodium nitroprusside	Sigma-Aldrich	Cat#228710; CAS: 13755-38-9
L-NAME	Sigma-Aldrich	Cat#N5751, CAS: 51298-62-5
Bradykinin	MedChemExpress	Cat#HY-P0206, CAS:

		58-82-2
Norepinephrine	MedChemExpress	Cat#HY-13715A, CAS: 329-56-6
4-Phenylbutyric acid (4-PBA)	MedChemExpress	Cat#HY-A0281, CAS: 1821-12-1
GSK2606414	MedChemExpress	Cat#HY-18072, CAS: 1337531-36-8
Tamoxifen	Sigma-Aldrich	Cat#T5648, CAS: 10540-29-1
[U-^{13}C] glucose	Cambridge Isotope Laboratories	Cat#CLM-1396
Mounting Medium with DAPI	Abcam	Cat#ab104139
Critical commercial assays		
ChamQ Universal SYBR qPCR Master Mix	Vazyme	Cat#R711-02
HiScript III RT SuperMix for qPCR (+gDNA wiper)	Vazyme	Cat#R323-01
Q300 Metabolite Assay Kit	Metabo-Profile Corp	Cat#HY5023R
Hyperactive Universal CUT&TAG Assay Kit for Illumina Pro	Vazyme	Cat#TD904
Pierce Protein G Magnetic	Thermo Scientific	Cat#88848

Beads		
TRIzol	Invitrogen	Cat#15596018
Lipofectamine RNAiMAX	Invitrogen	Cat#13778150
MitoSOX Red	Invitrogen	Cat#M36008
DAF-FM	Invitrogen	Cat#D23844
DCFH-DA for ROS	Meilunbio	Cat#MA0219
EdU Kit	Meilunbio	Cat#MA0425
Endothelial Cell Medium	ScienCell	Cat#1001
Intracellular NAD(P)H Flow Cytometric Analysis Kit	AAT Bioquest	Cat#15296
Experimental models: Organisms/strains		
Mouse: C57BL/6J	Gempharmatech	Strain NO. N000013
Mouse: *Nr1h4*$^{f/f}$	Gempharmatech	Strain NO. T009517
Mouse: *Cdh5-CreERT2*	Gempharmatech	Strain NO. T052686
Mouse: *ob/ob*	Gempharmatech	Strain NO. T001461
Mouse: *db/db*	Gempharmatech	Strain NO. T002407
Mouse: *ApoE*$^{-/-}$	Gempharmatech	
Software and algorithms		
FlowJo version 10.4.2	FlowJo	https://www.flowjo.com
DSI Ponemah 6.x	DSI	https://www.datasci.com/products/software/ponemah

Labchart 8.0	ADInstruments	https://www.adinstruments.com/support/labchart
GraphPad Prism 9.0	GraphPad Software	https://www.graphpad.com/scientific-software/prism/
ImageJ	NIH	https://imagej.nih.gov/ij/
SPSS 27.0	IBM SPSS	https://www.ibm.com/hk-en/products/spss-statistics
ZEN Microscopy	Zeiss	https://www.zeiss.com/microscopy/en/home.html
R studio	RStudio	https://www.r-project.org/
OmicStudio	LC-BIO	https://www.omicstudio.cn/tool
Adobe Illustrator	Adobe	https://www.adobe.com/products/illustrator.html
Others		
Normal chow diet	Tropic Animal Feed High-tech	Cat#TP23522
Rodent diet with 60% kcal% fat	Tropic Animal Feed High-tech	Cat#TP23400
HFD for atherosclerosis (Paigen diet)	Tropic Animal Feed High-tech	Cat#TP28640

Human study protocol

This study was conducted in accordance with the Declaration of Helsinki. All study participants were recruited after providing informed consent with approval by the Ethics Committee of Qilu Hospital of Shandong University (KYLL-202310 (YJ) -040). Human arterioles in omental adipose tissue and serum were obtained from Division of Bariatric and Metabolic Surgery, Department of General Surgery, Qilu Hospital of Shandong University.

We recruited participants with obesity, defined as BMI ≥ 30 kg/m^2, without hypertension (BP ≥ 140/90 mmHg). All individuals were carefully screened and excluded if they had used antihypertensives, insulin, GLP-1 receptor agonists, or any other medications known to affect metabolism or vascular function within the 3 months preceding their participation in the study. Metabolic syndrome criteria: (1) increased waist circumference (≥ 102 cm in male, ≥ 88 cm in female); (2) high blood pressure (BP ≥ 130/85 mmHg) or receiving anti-hypertensive therapy; (3) impaired fasting blood glucose (FBG ≥ 5.6 mmol/L); (4) increased fasting triglyceride (TG ≥ 1.7 mmol/L); (5) decreased high-density lipoprotein cholesterol (HDL-C < 1.04 mmol/L in male, < 1.29 mmol/L in female). Participants were grouped either into those with metabolically healthy obesity (MUO), or metabolically healthy obesity (MHO), defined as obese individuals meeting ≤ 1 of the metabolic syndrome criteria with exception of increased waist circumference. A flow diagram illustrating the human study protocol is shown in **Figure 1A**.

Wire myograph

Fresh human arterioles (150–250 μm) in omental adipose tissue and mouse 3rd-4th mesenteric arteries (150–250 μm) were isolated in ice-cold Krebs solution. The whole procedure was done carefully to avoid stretch-induced injury to the endothelium. In some experiments, artery rings (about 1.5mm in length) were incubated with different BAs or agonists for 12 h (GW4064 10 μM, INT-777 10 μM, TCDCA 50 μM, CDCA 50 μM, GCDCA 50 μM, DCA 50 μM, LCA 50 μM, TUDCA 50 μM, TβMCA 50 μM). Artery rings were mounted on an Automated Multi Wire Myograph System (Cat#630MA, DMT, DK). Briefly, 2 tungsten wires (40 μm diameter) were carefully inserted into the arterial lumen, and fixed to a force transducer and a micrometer respectively. Arteries were bathed in an oxygenated (95% O_2 and 5% CO_2) organ chamber containing 37 °C Krebs solution and set to the baseline circumference. After stabilizing for 30 min, arteries viability was tested using a potassium-rich solution (60 mM). Endothelium-dependent vasodilation was induced by cumulative concentration of ACH (Cat#A6625, Sigma-Aldrich, USA) in norepinephrine (NE, 10^{-5} mol/L, Cat# HY-13715A, MCE, USA) pre-contracted segments with or without L-NAME (30 min incubation, 10^{-4} M, Cat#N5751, Sigma-Aldrich, USA). Endothelium-dependent vasodilation was also induced by cumulative concentration of BK (Cat#HY-P0206, MCE, USA). Cumulative concentration response curves in response to SNP (Cat#228710, Sigma-Aldrich, USA) were used to assess the endothelium-independent relaxation of arteries.

Mouse models

Specific-pathogen-free (SPF) C57BL/6J mice (Strain NO. N000013), *Nr1h4*$^{flox/flox}$ mice (background: C57BL/6J, Strain NO. T009517), *Cdh5-CreERT2* mice (background: C57BL/6J, Strain NO. T052686), *ob/ob* (background: C57BL/6J, Strain NO. T001461), *db/db* (background: C57BLKS/J, Strain NO. T002407), and *ApoE*$^{-/-}$ (background: C57BL/6J, Strain NO. T001458) were purchased from Gempharmatech (Nanjing, China). Transgenic mice and wildtype mice were bred and maintained within sterile isolators on a 12 h–12 h light-dark cycle with ambient temperature of 20–26 °C, humidity of 40%–70%, and ad libitum access to food and water. The chow diet (TP23522, Trophic Animal Feed High-tech, China) was used in animal experiments. This study was approved by the Animal Ethics Committee of the Qilu Hospital of Shandong University (DWLL-2020-121). Animals were randomly assigned to each group. Body weight was measured and compared at the starting timepoint to ensure that there is no systematic bias in the allocation of animals to different experimental conditions. The sample size was determined empirically based on anticipated fold change from results obtained from similar experimental measurements or previous studies. No samples have been intentionally omitted from the *in vivo* study unless attributed to the death of a mouse before reaching the final time point. A double-blind strategy was implemented in the studies with mice to minimize the potential for bias in data collection. Caretakers fed mice and measured body weight and were unaware of the treatment conditions. The researcher conducting the final assessments and mouse dissection was also unaware of the treatment group to which each mouse belonged to prevent an unintentional influence on the study.

Animal experiment 1: DIO mice model

Mice (8-weeks-old C57BL/6J, male, n=8) were fed with HFD (TP23400, Trophic Animal Feed High-tech, China) for 12 weeks and euthanized for myograph experiments.

Animal experiment 2: VSG on EC-specific FXR knockout mice

For EC-specific FXR knockout, *Nr1h4*$^{f/f}$ mice were crossed with *Cdh5-CreERT2* mice. Male 6-weeks-old *Nr1h4*$^{f/f}$-*Cdh5-CreERT2*$^{-/-}$ (*Nr1h4*$^{f/f}$, n=16) and *Nr1h4*$^{f/f}$-*Cdh5-CreERT2*$^{+/-}$ (*Nr1h4*$^{\Delta EC}$, n=16) were intraperitoneally injected with tamoxifen (50 mg/kg, Cat#T5648, Sigma-Aldrich) for 5 consecutive days. 8-weeks-old *Nr1h4*$^{f/f}$ and *Nr1h4*$^{\Delta EC}$ mice were fed with HFD for 12 weeks and then randomly divided into 4 groups treated with sham or VSG: (1) *Nr1h4*$^{f/f}$ +Sham; (2) *Nr1h4*$^{f/f}$ +VSG; (3) *Nr1h4*$^{\Delta EC}$+Sham; (4) *Nr1h4*$^{\Delta EC}$+VSG. After 24-hour circadian BP telemetry, 28-weeks-old mice were euthanized for myograph experiments.

Animal experiment 3: VSG on EC-specific FXR overexpression mice

Mice (8-weeks-old C57BL/6J, male, n=32) were fed with HFD for 8 weeks, then randomly divided into 2 groups. 16 mice were intravenous treated with *ICAM2* promoter-driven AAV9-*Nr1h4* (1x10^{12} vg/ml) for EC-specific FXR overexpression. Another 16 mice were intravenous treated with *ICAM2* promoter-driven AAV9-Ctrl (1x10^{12} vg/ml). 20-weeks-old AAV9-Ctrl and AAV9-*Nr1h4* mice were randomly divided into 4 groups treated with sham or VSG: (1) AAV9-Ctrl +Sham; (2) AAV9-Ctrl +VSG; (3) AAV9-*Nr1h4*+Sham; (4) AAV9-*Nr1h4*+VSG. After 24-h

circadian BP telemetry, 28-weeks-old mice were euthanized for myograph experiments.

Animal experiment 4: TCDCA treatment on EC-specific FXR knockout mice

Male 6-weeks-old *Nr1h4*$^{f/f}$ (n=16) and *Nr1h4*$^{\Delta EC}$ (n=16) were intraperitoneally injected with tamoxifen (50 mg/kg, Cat#T5648, Sigma-Aldrich) for 5 consecutive days. 8-weeks-old *Nr1h4*$^{f/f}$ and *Nr1h4*$^{\Delta EC}$ mice were fed with HFD for 12 weeks and then randomly divided into 4 groups treated with vehicle or TCDCA for 4 weeks (20 mg/kg/day, intraperitoneal injection): (1) *Nr1h4*$^{f/f}$ +vehicle; (2) *Nr1h4*$^{f/f}$ +TCDCA; (3) *Nr1h4*$^{\Delta EC}$+vehicle; (4) *Nr1h4*$^{\Delta EC}$+TCDCA. After 24-hour circadian BP telemetry, 28-weeks-old mice were euthanized for myograph experiments.

Animal experiment 5: TCDCA treatment on *ob/ob* mice

8-weeks-old male *ob/ob* mice (n=16) were divided into 2 groups and treated with vehicle or TCDCA for 4 weeks (20 mg/kg/day, intraperitoneal injection): (1) *ob/ob*+vehicle; (2) *ob/ob*+TCDCA.

Animal experiment 6: TCDCA treatment on *db/db* mice

8-weeks-old male *db/db* mice (n=16) were divided into 2 groups and treated with vehicle or TCDCA for 4 weeks (20 mg/kg/day, intraperitoneal injection): (1) *db/db*+vehicle; (2) *db/db*+TCDCA.

Animal experiment 7: TCDCA treatment on *ApoE*$^{-/-}$ mice

8-weeks-old male *ApoE*$^{-/-}$ mice (n=16) were fed with HFD (TP28640, Trophic Animal Feed High-tech, China) for 8 weeks, then randomly divided into 2 groups treated with

vehicle or TCDCA for 4 weeks (20 mg/kg/day, intraperitoneal injection): (1) vehicle; (2) TCDCA.

Statistics

All raw data and results were interpreted in a blinded fashion. Statistical analyses were performed using R v4.4.1, SPSS v27.0, and GraphPad Prism v9.0 software (GraphPad Software, USA). For statistical comparisons, the data distribution was determined using the Shapiro-Wilk normality test. Normally distributed data are presented as mean ± standard deviations (SD) while non-normally distributed data are presented as median and interquartile range. Homogeneity of variance was evaluated using with F test (for Student *t* test) or Bartlett's test (for one-way ANOVA). The statistical differences between groups were assessed as follows: (1) For 2 independent groups, demonstrating normal distribution with homogeneous variances, analysis was performed using the two-tailed Student *t* test. In scenarios of heterogeneity in variances, the two-tailed Student *t* test incorporating Welch correction was applied. For distributions deviating from normality (including $n \leq 4$), the Mann-Whitney U test was applied. (2) For data sets involving more than 2 independent groups, with a singular factor under normal distribution, the analysis involved the use of one-way ANOVA for homogenous variances, complemented by Tukey's multiple comparisons test for posthoc insights. In cases of variance heterogeneity under normal distribution, the Brown-Forsythe ANOVA test was employed with Dunnett's T3 multiple comparisons test. For repeated measures, one-way ANOVA with Greenhouse-Geisser correction was used followed by with Tukey's post-hoc test. For groups not

conforming to normal distribution, the Kruskal-Wallis test was used with adjustment by multiple testing using FDR correction (Benjamini-Hochberg method). (3) For data sets involving more than 2 independent group with 2 factors, two-way ANOVA followed by Tukey multiple comparison tests was used. (4) Correlations were examined by the spearman rank correlation coefficients with adjustment by multiple testing using FDR correction (Benjamini-Hochberg method). The statistical details of experiments can be found in this paragraph and in the main and supplemental figure legends.

Mice vertical sleeve gastrectomy (VSG) and sham surgery

VSG was performed in DIO mice using isofluorane anaesthesia. The lateral 80% of the stomach was excised leaving a tubular gastric remnant in continuity with the oesophagus superiorly and the pylorus and duodenum inferiorly. The sham procedure involved analogous isolation of the stomach followed by manually applying pressure with blunt forceps along a vertical line between the esophageal sphincter and the pylorus. Mice consumed liquid diet for the first 3 post-operative days, and were re-introduced to HFD.

Glucose tolerance test (GTT)

A glucose tolerance test was performed after overnight fasting. Glucose levels were measured at 0, 15, 30, 60 and 120 min after intraperitoneal injection of glucose (1.2 g/kg) using a blood glucose meter (Accu-Chek Performa, Roche).

Metabolic profiling of serum samples from patients

The targeted metabolomics analysis on serum samples (50 μL) was conducted using

the Q300 Metabolite Assay Kit provided by Metabo-Profile Biotechnology Co., Ltd (Shanghai, China). After samples preparation according to the manufacture's instruction, the Q300 plate was sealed for ultraperformance liquid chromatography coupled with tandem massspectrometry (UPLC-MS/MS, ACOUITY UPLC-Xev, TQ-S, Waters, Milford, MA, USA) analysis according to a protocol previously established.[1] All chromatographic separations were performed with an ACQUITY BEHC18 column (1.7 wm, 100 mm x 2.1 mm internal dimensions; Waters, Milford, MA, USA). The mobile phase consisted of 0.1% formic acid in LC-MS grade water (mobile phase A) and 0.1% formic acid in LC-MS grade acetonitrile (mobile phase B) run at a flow rate of 0.3 ml/min. Gradient elution with the mobile phase at a flow rate of 0.45 ml/min: 0-1 min (5% B), 1-5 min (5-25% B), 5-15.5 min (25-40% B), 15.5-17.5 min (40-95%B),17.5-19 min (95% B),19-19.5 min (95-5% B), and 19.6-21 min (5% B). The column was maintained at 45°C and the injection volume of all samples was 5 μL. The mass spectrometer was operated in negative ion mode with a capillary voltage of 1.2 kV. The source temperature is 150 °C, and the desolyation gas temperature is 550 °C. The data were collected with multiple reaction monitor (MRM), and the cone andcollision energy used the optimized settings from QuanOptimize application manager (Waters). Samples were analyzed randomly by generating random numbers to reduce the error introduced by the instrument. Quality control (QC) samples, prepared from pooled samples, were analyzed every 12 samples across the entire sample set to assess instrument stability and consistency in sample processing. The raw data from UPLC-MS/MS were processed using

QuanMET software (v2.0, Metabo-Profile, Shanghai, China) for peak integration, calibration, and quantification of each metabolite.

Telemetry for BP monitor

Continuous BP was measured using implanted radiotelemetry probes (Data Sciences International, cat# HD-X11, New Brighton, MN, USA). Mice were anesthetized with isoflurane inhalation. The left carotid artery was surgically exposed, and the radiotelemetry probe catheter was carefully advanced approximately 1 cm into the arterial lumen and secured in place with sutures. The body of the telemetry probe was placed subcutaneously on the left flank. Mice were allowed to recover for a minimum of 7 days post-surgery before blood pressure data were continuously recorded for a 24-hour period using receivers and a data acquisition system

Cell culture and experiments

The procedures for the isolation of human-derived primary cells were approved by the ethics committees of Qilu Hospital of Shandong University. HUVECs were isolated from umbilical cords. The umbilical cord was washed with PBS 3 times, clamped at both ends, filled with 0.25% trypsin, and then placed at 37 °C. After 15 min, the umbilical cord was gently kneaded to digest endothelial cells, and then rinsed with endothelial cell medium (ECM, Cat#1001, ScienCell) to collect endothelial cells. The HUVECs at passages 4 to 6 were used for experiments. Cell cultures were maintained at 37 °C in a humidified atmosphere containing 5% CO_2. We performed siRNA transfection experiments using lipofectamine RNAiMAX (Cat#13778150, Invitrogen)

according to manufacturer's guidelines. siRNAs were purchased from GenePharma (China) as shown in Supplementary Table S5.

RNA-sequencing

otal RNA was isolated from HUVECs using TRIzol reagent (Cat#15596018, Invitrogen, USA) according to the manufacturer's instructions. The amount and purity of isolated RNA were quantified using a NanoDrop ND-1000 spectrophotometer (NanoDrop, Wilmington, DE, USA). RNA integrity was assessed using a Bioanalyzer 2100 system (Agilent, CA, USA), and samples with an RNA Integrity Number (RIN) > 7.0 were used for library preparation. RNA integrity was further confirmed by electrophoresis on a denaturing agarose gel. For library preparation, Poly(A)+ RNA was purified from 1μg total RNA using Dynabeads Oligo $(dT)_{25}$ (Invitrogen, Cat#61005, USA) using 2 rounds of purification. The purified poly(A)+ RNA was then fragmented using the Magnesium RNA Fragmentation Module (NEB, Cat#e6150, USA) by incubating at 94 ◦ C for 5–7 minutes in the provided buffer. Fragmented RNA was reverse transcribed into first-strand cDNA using SuperScript™ II Reverse Transcriptase (Invitrogen, Cat#1896649, USA). Second-strand cDNA was subsequently synthesized using E. coli DNA Polymerase I (NEB, Cat#m0209, USA), RNase H (NEB, Cat#m0297, USA), and dUTP Solution (Thermo Scientific, Cat#R0133, USA), generating U-labeled double-stranded cDNA. An A-base is then added to the blunt ends of each strand, preparing them for ligation to the indexed adapters. Each adapter contains a T-base overhang for ligating the adapter to the A-tailed fragmented DNA. Single- or dual-index adapters are ligated to the fragments,

and size selection was performed with AMPureXP beads. After the heat-labile UDG enzyme (NEB, Cat#m0280, USA) treatment of the U-labeled second-stranded DNAs, the ligated products are amplified with PCR by the following conditions: initial denaturation at 95℃ for 3 min; 8 cycles of denaturation at 98℃ for 15 sec, annealing at 60℃ for 15 sec, and extension at 72℃ for 30 sec; and then final extension at 72℃ for 5 min. The average insert size for the final cDNA library was 300 ± 50 bp. Libraries were sequenced on an Illumina NovaSeq 6000 platform to generate 2×150 bp paired-end reads (PE150). Sequencing was performed following the vendor's recommended protocol by LC-Bio Technology CO., Ltd. (Hangzhou, China).

Reverse transcription-quantitative polymerase chain reaction (RT-qPCR)

Total RNA was isolated and purified using TRIzol reagent and reverse-transcribed to cDNA using HiScript III RT SuperMix for qPCR (Cat#R323, Vazyme, China). qPCR was carried out using ChamQ Universal SYBR qPCR Master Mix (#R711, Vazyme, China). Primers were purchased from BioSune Biotech (China) as shown in Supplementary Table S3.

Cleavage Under Targets and Tagmentation-Sequencing (CUT&TAG)

CUT&Tag was outsourced to Shanghai Jiayin Biological Technology Co., Ltd. (Shanghai, China). CUT&TAG assays were performed using the Hyperactive Universal CUT&TAG Assay Kit for Illumina Pro (Cat#TD904, Vazyme, China) according to the manufacturer's instructions. Briefly, cells were collected and mixed with 10 μL of activated Concanavalin A-coated magnetic beads. The cell-bead

mixture was incubated at room temperature for 10 minutes to bind the cells to the beads. Bead-bound cells were resuspended in 100 μL of buffer containing primary antibody FLAG (1:50, Cat#F1804, Sigma-Aldrich, USA), PHB1 (Invitrogen, Cat# MA5-12858), FXR (Invitrogen, Cat#417200) or normal IgG (Cat#12-371, Sigma-Aldrich, USA). The mixture was incubated overnight at 4 °C. After removing the primary antibody buffer using a magnetic rack, cells were incubated with a secondary antibody goat for 60 min at room temperature. The Hyperactive pA-Tn5 transposase was prepared and incubated with cells for 1 h at room temperature. Next, the cells were resuspended in the Tagmentation buffer and incubated at 37 °C for 1 h. DNA was purified using phenol-chloroform-isoamyl alcohol extraction and ethanol precipitation. The libraries were amplified by PCR with a universal i5 and a uniquely barcoded i7 primer. Size analysis of the finished library was conducted using the Agilent 4200 TapeStation system (Agilent Technologies, Santa Clara, CA, USA). Finally, sequencing was performed with the Illumina NovaSeq 6000 platform and 150 bp paired-end reads were generated for the subsequent analysis.

Chromatin immunoprecipitation (ChIP)

ChIP assays were performed using the SimpleChIP Enzymatic Chromatin IP kit (Magnetic Beads) (Cat#9005, Cell Signaling Technology, USA) according to the manufacturer's protocol. In brief, HUVECs with different treatments were incubated with 1% fresh paraformaldehyde at room temperature for 10 min to crosslink the histone/transcription factor complexes with DNA, followed by 0.1% glycine incubation at room temperature for 5min. The nuclei pellets were digested with

Micrococcal Nuclease at 37°C for 20 min, followed by sonication. After centrifugation, the chromatin was immunoprecipitated with antibodies against FXR (1:50 dilution, Cat#417200, Invitrogen, USA), PHB1 (1:50 dilution, Cat# MA5-12858, Invitrogen, USA), or normal mouse IgG (Cat#12-371, Sigma-Aldrich, USA) overnight with gentle rotation. The protein/DNA complexes were immunoprecipitated by 30 μL ChIP grade protein G magnetic beads with rotation for 2 h at 4 °C, followed by three washes in low-salt buffer and one wash in high-salt buffer, and elution at 65 °C for 30min. The eluted protein-DNA complexes were reversed with proteinase K at 65 °C for 2 h. The DNA was purified and then amplified by quantitative real-time PCR. The ChIP-qPCR data were reported as percentage of input, which can be calculated by the formula:

% of input =2^((Ct(input)-log2(dilution factor))-Ct(ChIP))*100%.

The input sample used was 10% of the DNA amount, and thus the dilution factor is 10. Ct (Input) is the quantification cycle of the undiluted input sample, and Ct (ChIP) is the quantification cycle of the immunoprecipitated sample.

^{13}C labeled glucose tracing

HUVECs were cultured with glucose-free MEM supplemented with 5×10^{-3} M [U-^{13}C] glucose (Cambridge Isotope Laboratories, Cat#CLM-1396) for 24 h. After the tracer experiment, medium was aspirated, and cells were washed twice with PBS. The live viable cells were calculated. The 80% methanol was added to each tube followed by sonicate and centrifugation. The Metabo-Profile Biotechnology Co., Ltd (Shanghai, China) conducted UHPLC-MS analysis and the abundance of metabolites was

quantified following established protocols.

Immunoprecipitation (IP)

HUVECs were washed with cold PBS and lysed with lysis buffer supplemented with 1% protease inhibitor cocktail. The whole cell lysates were incubated with specific antibody and Protein G Magnetic Beads overnight at 4 °C. Subsequently, the immunocomplexes were washed with lysis buffer for 3 times at 4 °C and eluted with loading buffer at 100 °C for 5 min. Protein sample was resolved by SDS-PAGE and immunoblot. For screening the binding proteins of FXR, the LC-MS/MS analysis was carried out by Sinotech Genomics (Shanghai, China). Finally, we screened out the substrate proteins that could bind to FXR according to the score and the mass of detected proteins.

Mitochondrial ROS Measurement

To evaluate mitochondrial ROS production, HUVECs seeded in confocal dishes were treated as specified, then stained with MitoSOX Red probe (Cat#M36008, Invitrogen, USA) for 30 min at 37 °C in darkness, following the manufacturer's instructions. Images were acquired using a confocal microscope camera (Zeiss LSM 900, Zeiss, Germany) and further analyzed by ImageJ or ZEN software.

To evaluate mitochondrial ROS production in arteries, isolated arteries were cannulated and intraluminally infused with the MitoSOX Red probe (Cat#M36008, Invitrogen, USA) mounted on a pressurized myograph (Cat#114P, DMT, Denmark). Arteries were exposed to the MitoSOX Red probe for 1 h at 30 mmHg pressure. Images were acquired using microscope camera (Zeiss, Germany) and further

analyzed by ImageJ or ZEN software.

Echocardiography

Transthoracic echocardiography was performed at the end of the experiment using the Vevo 3100 Imaging System (VisualSonics Inc.) to evaluate the cardiac function in mice. The mice were anesthetized with 2% inhaled isoflurane and placed on a heating platform at 37 °C. Left ventricular end-diastolic diameter and left ventricular end-systolic diameter were measured by M-mode echocardiography in the parasternal long-axis view, and FS and EF were calculated automatically using the imaging system.

Atherosclerotic lesion analysis

The atherosclerotic lesions in the aorta were quantified by *en face* analysis and aortic root cross-sectional measurement. For *en face* analysis, whole aorta was isolated and stained with 0.5% oil red O (ORO, Sigma, St. Louis, MO) for 30 min prior to visualization. The ORO-stained lesions were quantified as a percentage of the total aortic area using Image J software. For aortic root cross sectioning, the heart with attached aortic roots was embedded in Tissue-Tek OCT Compound, and serially sectioned at 5 μm through the aorta beginning at the origin of the aortic valve leaflets. Slides were stained with ORO and hematoxylin-eosin following the manufacturer's protocol.

NO Measurement

To evaluate NO production, HUVECs were treated as specified, then stained with DAF-FM (Cat#D23844, Invitrogen, USA) for 30 min at 37 °C in darkness, following

the manufacturer's instructions. Intracellular NO contents were verified by flow cytometry.

To evaluate NO production in arteries, isolated arteries were cannulated and intraluminally infused with the DAF-FM mounted on a pressurized myograph (Cat#114P, DMT, Denmark). Arteries were exposed to the DAF-FM probe for 1 h at 30 mmHg pressure, followed by 30 min at 60 mmHg during which treatments were added. Images were acquired using microscope camera (Zeiss, Germany) and further analyzed by ImageJ or ZEN software.

NAD(P)H Measurement

To evaluate NAD(P)H contents, HUVECs were treated as specified, Intracellular NAD(P)H contents were verified by flow cytometry using a Cell Meter Intracellular NADH/NADPH Flow Cytometric Analysis Kit (Cat#15296, AAT Bioquest, USA) according to the manufacturer's instructions.

Immunofluorescence (IF) staining

For *in vitro* studies, cells on the slides were washed and fixed with 4% paraformaldehyde solution at room temperature for 20 minutes and permeabilized with 0.5% Triton X-100 for 5 min. After blocking with 5% bovine serum albumin, the slides were incubated with FXR and PHB1 primary antibodies overnight at 4 °C. After three washes, the slides were incubated with secondary antibodies for 1 h at room temperature. After washing with PBS for 3 times, slides were mounted with Mounting Medium with DAPI (Cat#ab104139, Abcam) and sealed. Images were acquired using a confocal microscope camera (Zeiss LSM 900, Zeiss, Germany) and

further analyzed by ImageJ or ZEN software.

For the IF analysis of human and mice arteries, arteries were initially fixed using 4% paraformaldehyde overnight. They were then embedded in OCT and sectioned serially at a thickness of 5 μm. Blocking was performed with 5% bovine serum albumin for 60 minutes at room temperature. The sections were then incubated with primary antibodies overnight at 4 °C. After washing with PBS for 3 times, secondary antibodies were incubated with secondary antibodies for 1 h at room temperature. After washing with PBS for 3 times, sections were mounted with Mounting Medium with DAPI (Cat#ab104139, Abcam) and sealed. Images were acquired using a confocal microscope camera (Zeiss LSM 900, Zeiss, Germany) and further analyzed by ImageJ or ZEN software.

Figures and figure legends

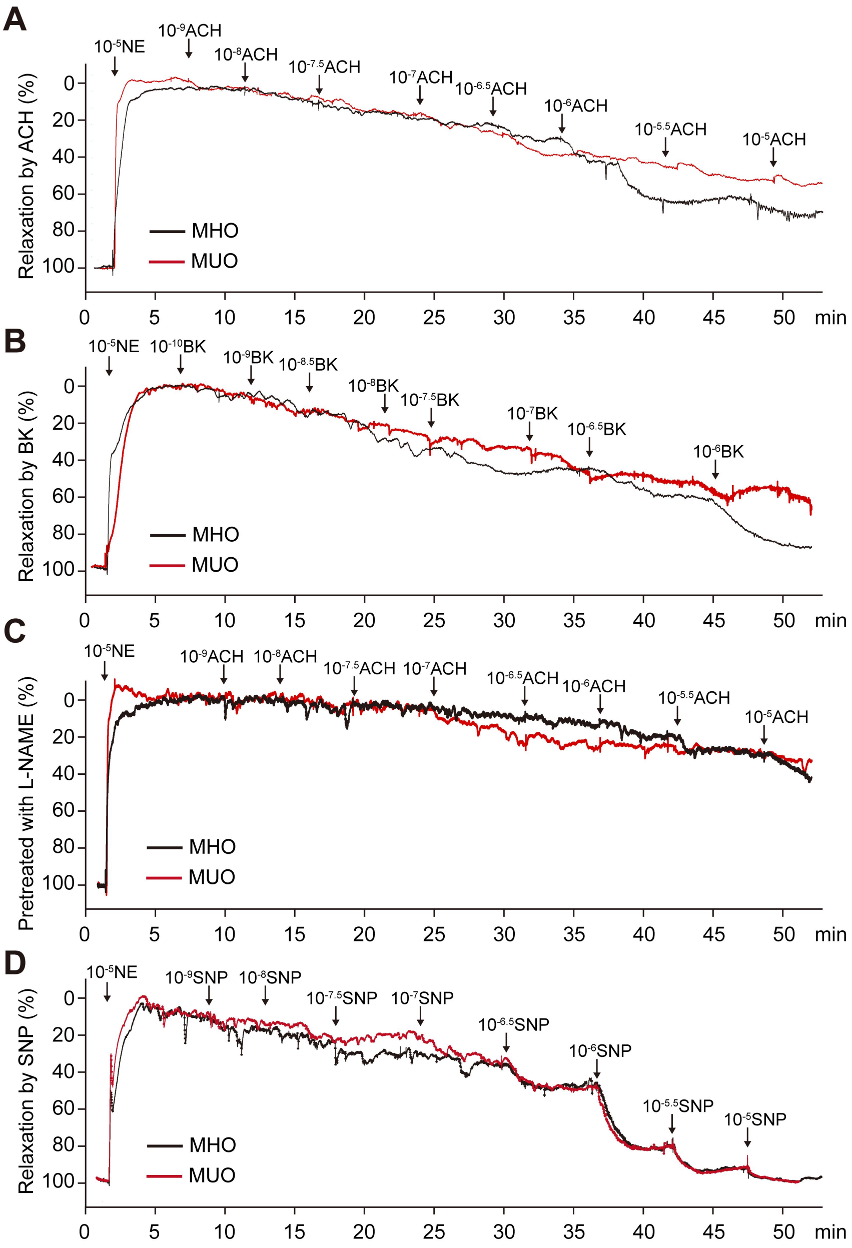

Figure S1 Representative Wire Myograph Force Traces of MHO or MUO Groups

(A-B) Endothelium-dependent vasodilation responses to cumulative concentrations of ACH (A) or BK (B) in human arterioles from MHO and MUO patients.

(C-D) Endothelium-independent vasodilation responses to cumulative concentrations of ACH following L-NAME pretreatment in human arterioles or to cumulative concentrations of SNP from MHO and MUO patients.

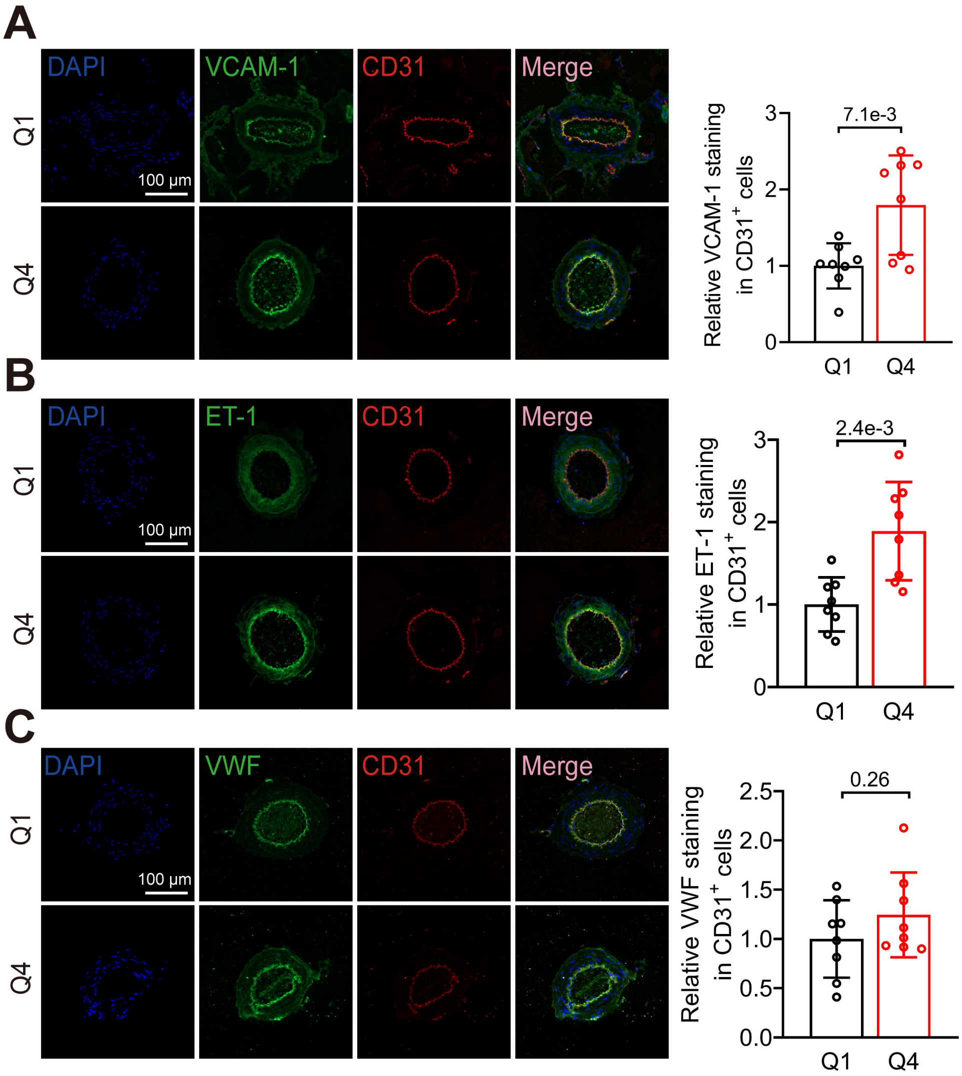

Figure S2 VCAM-1, ET-1, and VWF IF Staining of Human Arterioles

(A-C) Representative IF staining and quantitative analysis in arterioles from the Q1 and Q4 groups based on AUC-ACH levels (scale bar = 100 μm). Panels show staining for: VCAM-1 (green) and CD31 (red) in (A); ET-1 (green) and CD31 (red) in (B); and VWF (green) and CD31 (red) in (C). Quantitative analysis shows fluorescence intensity within $CD31^{+}$ cells for each marker (n=8).

Data are presented as mean ± SD. Statistical analysis was performed using Student *t* test (A-C)

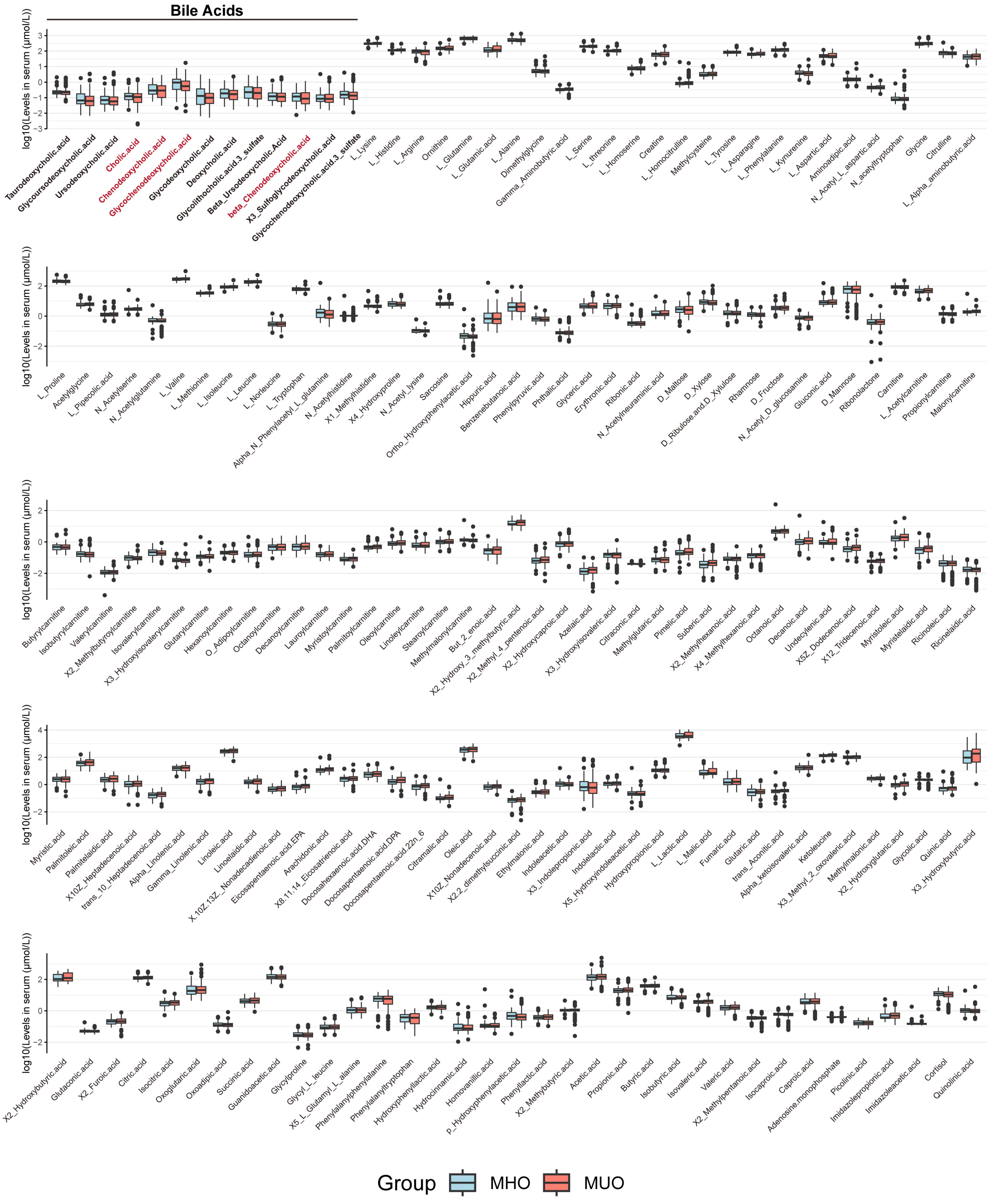

Figure S3 All 191 Metabolite levels in MHO and MUO Groups

Box plots showing the expression levels of all 191 detected metabolites in the MHO and MUO groups.

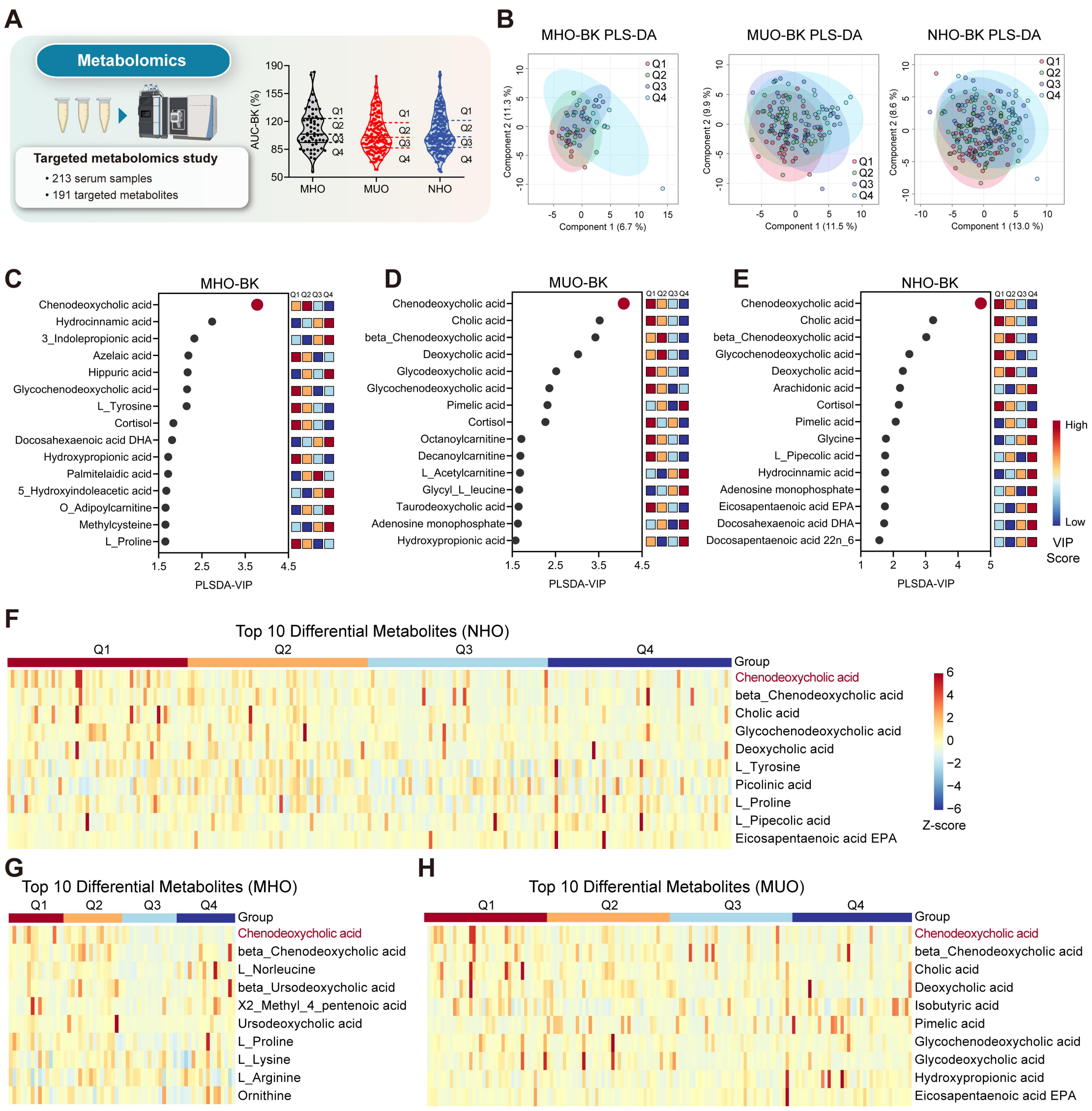

Figure S4 Metabolomics Reveals CDCA Associated with Obesity-induced ED Indicated by AUC-BK

(A) Metabolomics flow chart and schematic diagram for AUC-BK-IQR (Q1, Q2 Q3, Q4 groups) in the MHO group (n=62), the MUO group (n=151) or the whole NHO patients (n=213). AUC-BK data are derived from the experiments shown in Figure 1.

(B) PLS-DA showing the metabolites of Q1 -Q4 patients in the MHO, MUO, and NHO groups.

(C-E) VIP scores of the PLS-DA based on metabolic profile in the MHO (C), MUO (D), and NHO (E) groups.

(F-H) Heatmap of top 10 differential metabolites across Q1-Q4 groups in the NHO (F), MHO (G), and MUO (H) groups.

Data are presented as median with IQR. Statistical analysis was performed using the Kruskal–Wallis with Benjamini-Hochberg correction for multiple comparisons (F-H).

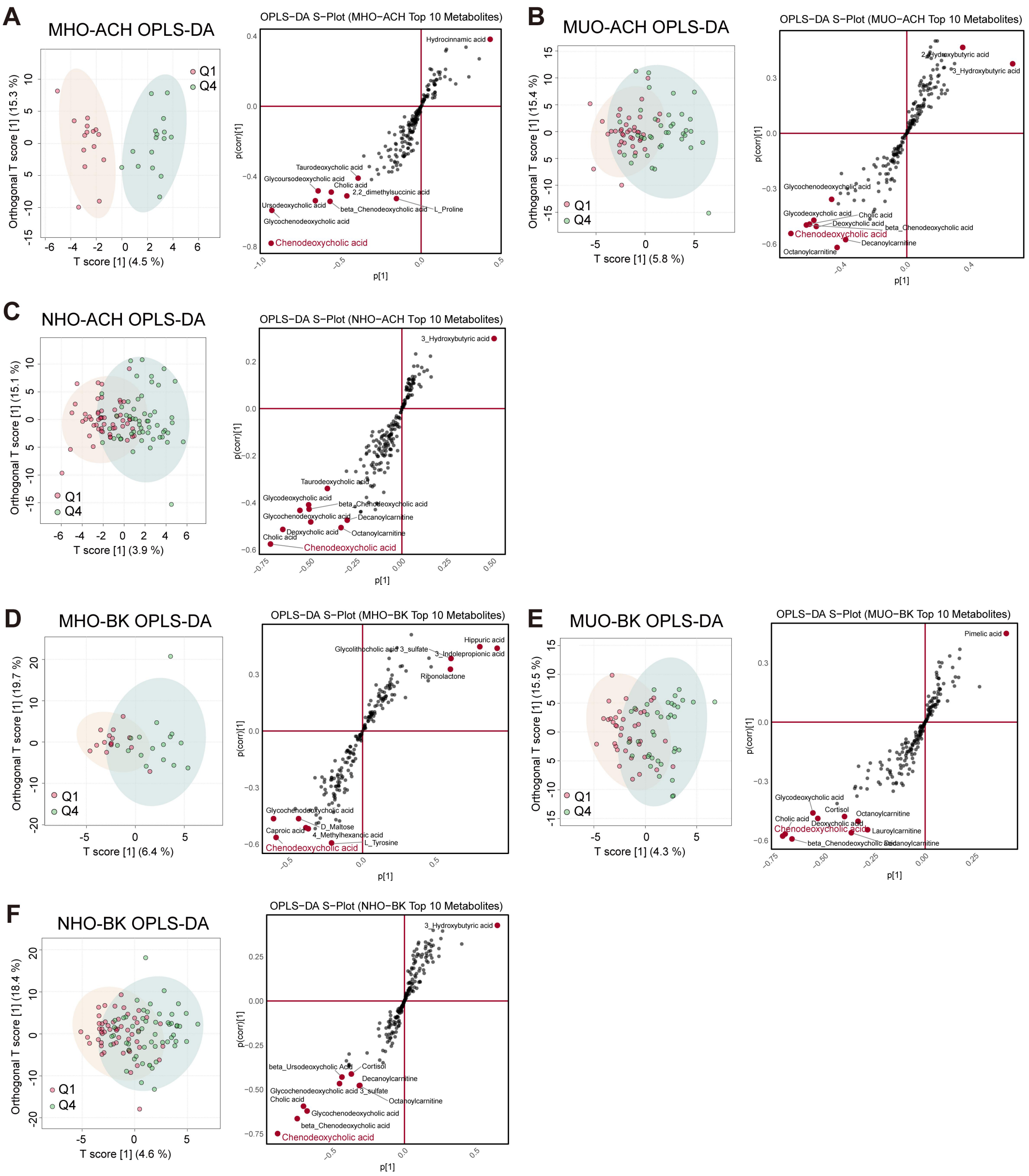

Figure S5 OPLS-DA comparing the Q1 and Q4 groups

(A-C) OPLS-DA based on AUC-ACH values and the corresponding S-plot comparing the Q1 and Q4 groups in MHO (A), MUO (B), and NHO (C) groups.

(D-F) OPLS-DA based on AUC-BK values and the corresponding S-plot comparing the Q1 and Q4 groups in MHO (D), MUO (E), and NHO (F) groups.

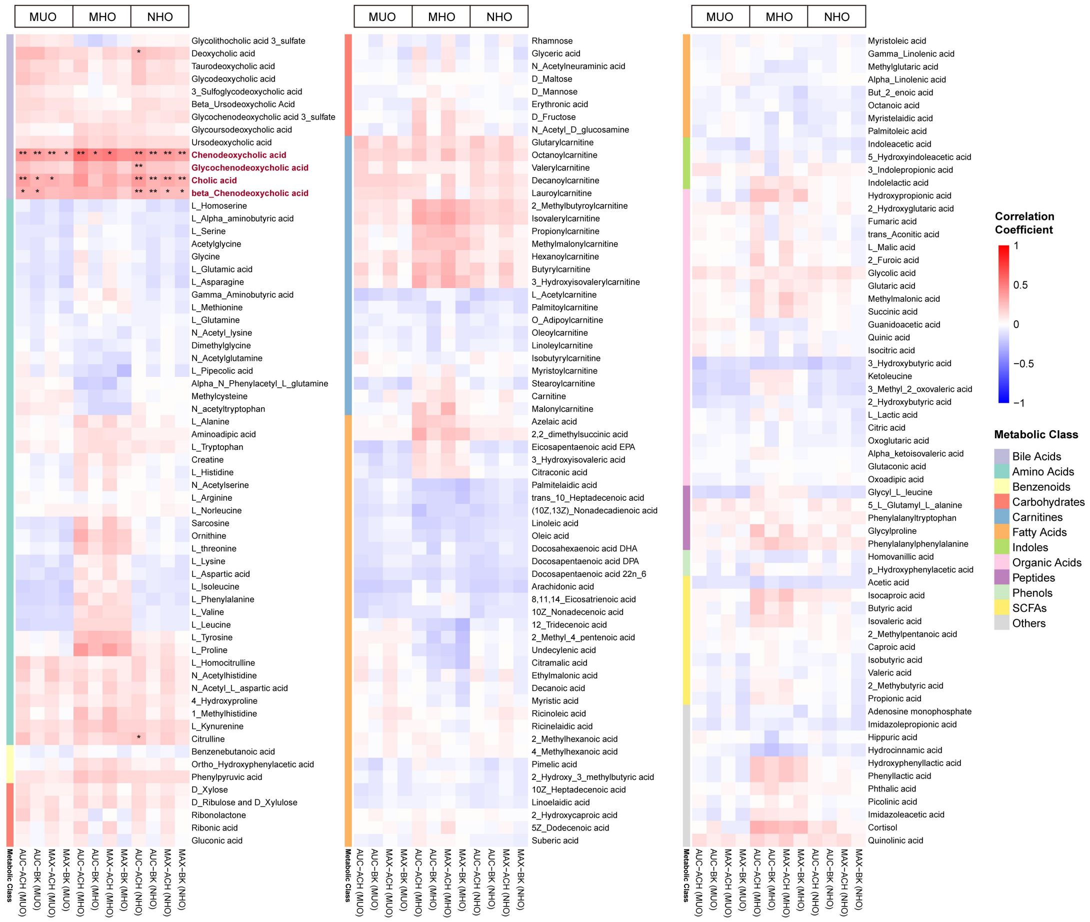

Figure S6 Correlation Analysis of Metabolite Profiles and Endothelial Function in MHO, MUO, and NHO Groups.

Heatmap visualizing the statistical significance of correlation coefficients between 191 metabolites and endothelial function parameters (AUC-ACH, MAX-ACH, AUC-BK, and MAX-BK) in the MHO, MUO, and NHO groups.

Statistical analysis was performed using the Spearman correlation with Benjamini-Hochberg correction for multiple comparisons. *adjusted $P<0.05$; **adjusted $P<0.01$; ***adjusted $P<0.001$.

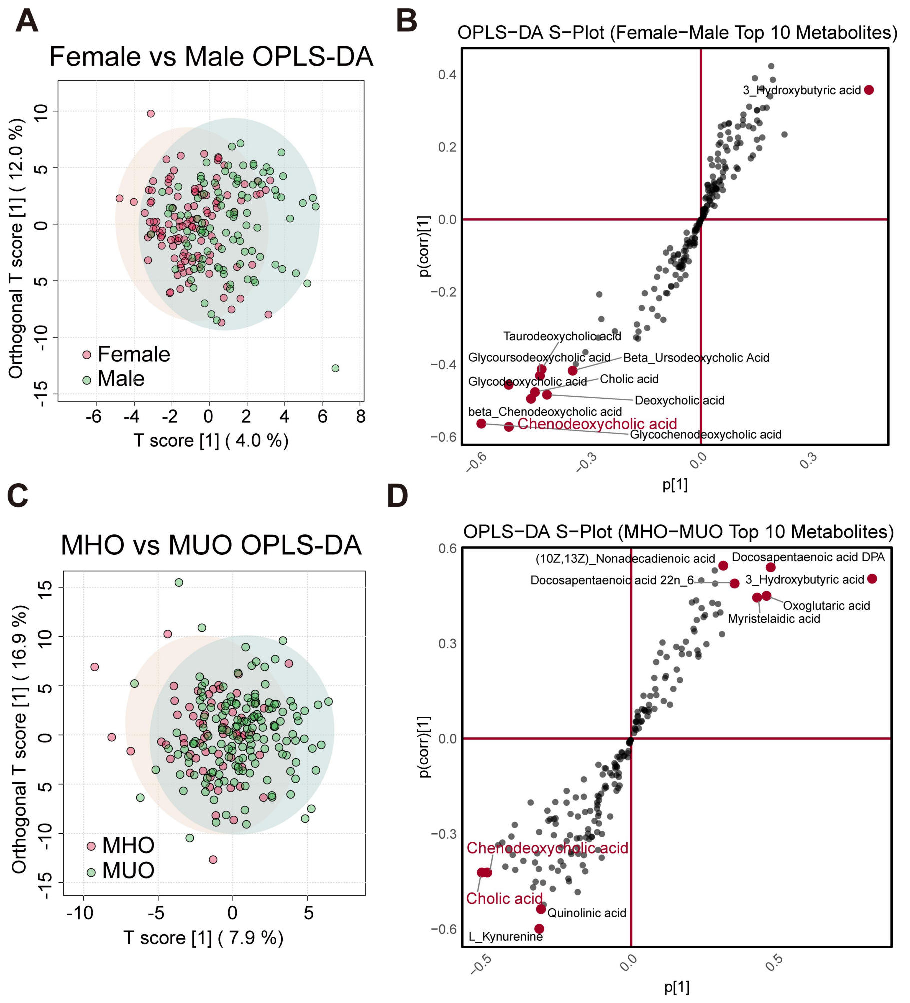

Figure S7 Comparisons of Metabolite Profiles Between Female and Male, and MHO and MUO Groups.

(A-B) OPLS-DA (A) and the corresponding S-plot (B) comparing the female (n=112) and male (n=101).

(C-D) OPLS-DA (C) and the corresponding S-plot (D) comparing the MHO (n=62) and MUO (n=151).

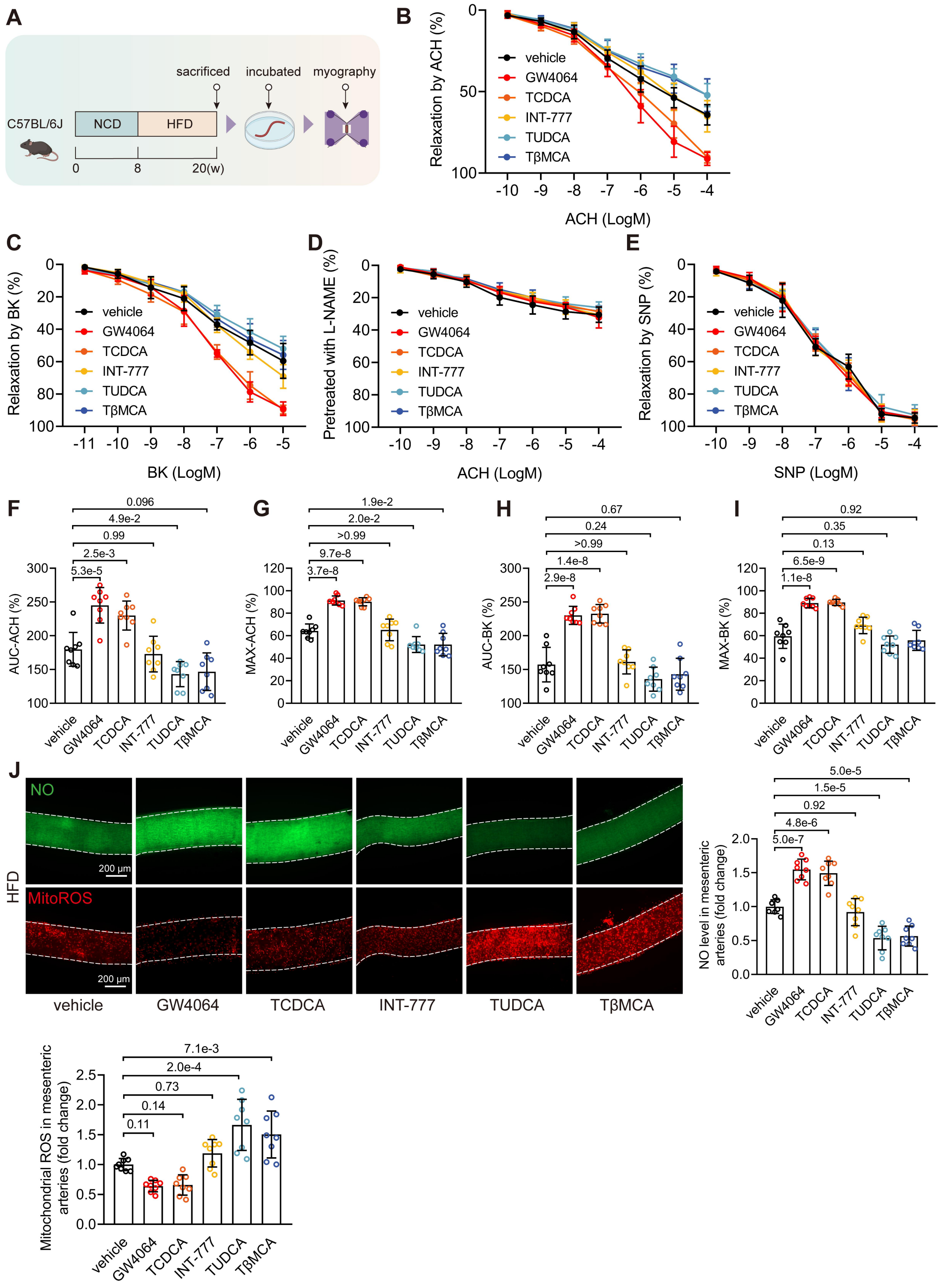
A
C57BL/6J
NCD
HFD
sacrificed
incubated
myography
0
8
20(w)
B
Relaxation by ACH (%)
ACH (LogM)
vehicle
GW4064
TCDCA
INT-777
TUDCA
TβMCA
C
Relaxation by BK (%)
BK (LogM)
D
Pretreated with L-NAME (%)
ACH (LogM)
E
Relaxation by SNP (%)
SNP (LogM)
F
AUC-ACH (%)
5.3e-5
2.5e-3
0.99
4.9e-2
0.096
G
MAX-ACH (%)
3.7e-8
9.7e-8
>0.99
2.0e-2
1.9e-2
H
AUC-BK (%)
2.9e-8
1.4e-8
>0.99
0.24
0.67
I
MAX-BK (%)
1.1e-8
6.5e-9
0.13
0.35
0.92
J
NO
MitoROS
HFD
200 μm
vehicle
GW4064
TCDCA
INT-777
TUDCA
TβMCA
NO level in mesenteric arteries (fold change)
5.0e-7
4.8e-6
0.92
1.5e-5
5.0e-5
Mitochondrial ROS in mesenteric arteries (fold change)
0.11
0.14
0.73
2.0e-4
7.1e-3

Figure S8 TCDCA Rescues Obesity-induced ED in *Ex vivo* DIO Mice Mesenteric Arteries

(A) Mice experiment flow chart. Mesenteric artery rings were divided into equal segments and incubated with different BAs or agonists (GW4064 10 μM, INT-777 10 μM, TCDCA 50 μM, TUDCA 50 μM, TβMCA 50 μM) for 12 h before assessment by wire myograph.

(B-C) Endothelium-dependent vasodilation to cumulative concentration of ACH (B) or BK (C) in DIO mice mesenteric arteries pretreated with different agonists or BAs (n=8).

(D-E) Endothelium-independent vasodilation to cumulative concentration of ACH pretreated with L-NAME (D) or to cumulative concentration of SNP (E) in DIO mice mesenteric arteries pretreated with different agonists or BAs (n=8).

(F-I) Endothelium-dependent vasodilation exhibited by AUC-ACH (F), MAX-ACH (G), AUC-BK (H), and MAX-BK (I) (n=8).

(J) Representative images of mitochondrial ROS and NO staining in DIO mice mesenteric arteries pretreated with different agonists or BAs (scale bar = 200 μm), along with quantified ROS and NO levels normalized to the vehicle group (n=8).

Data are presented as mean ± SD. Statistical analysis performed using repeated measures one-way ANOVA with Greenhouse-Geisser correction followed by Tukey's multiple comparisons test (F-J).

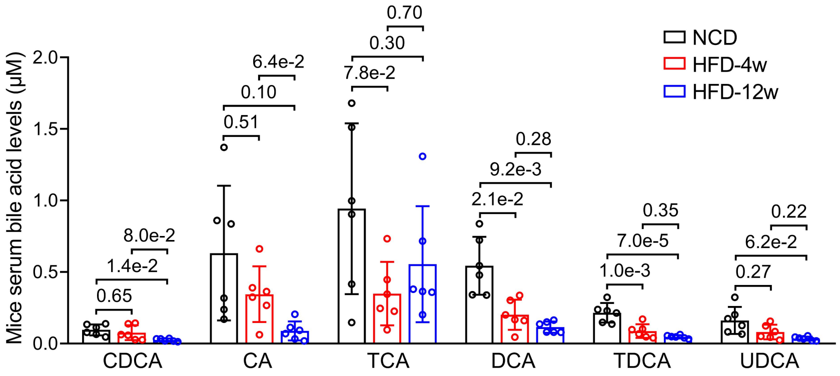

Figure S9 Targeted BA Metabolomics Profiles in DIO Mice.

Targeted BA metabolomics analysis was performed on serum samples from mice fed NCD, HFD for 4 weeks, and HFD for 12 weeks. This figure presents the profiles of the quantified BAs across these experimental groups.

Data are presented as mean ± SD. Statistical analysis was performed using one-way ANOVA followed by Tukey's post-hoc test or Brown-Forsythe ANOVA test followed by Dunnett's T3 post-hoc test.

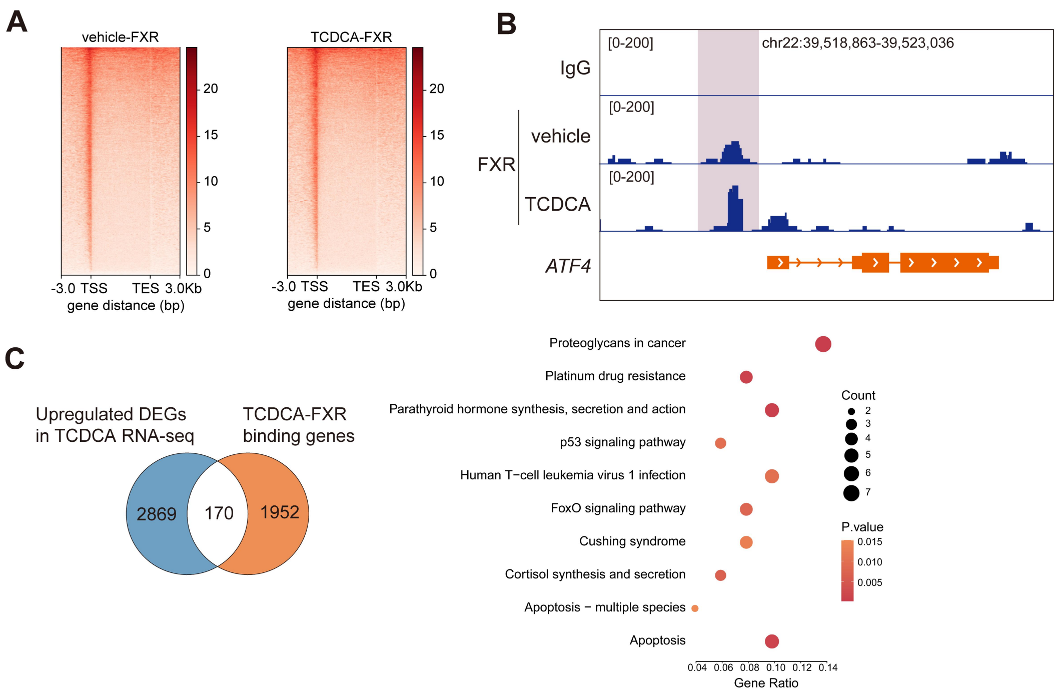

Figure S10 Combined Analysis of CUT&TAG and RNA-seq

(A) Heatmap showing normalized intensity profiles across regions spanning 3 kb upstream of the TSS, through the gene body, to 3 kb downstream of the TES for FXR-bound genes in HUVECs treated with TCDCA (50 μM, 24 h) or vehicle.

(B) IGV analysis representing IgG and FXR peaks at the *ATF4* loci in HUVECs treated with TCDCA (50 μM, 24 h) or vehicle.

(C) Venn plot (left) identified 170 common genes between 3039 significantly upregulated DEGs and 2122 FXR binding genes in promoter regions in HUVECs treated with TCDCA (50 μM, 24 h). Bubble plot showing significantly enriched KEGG pathways among the common genes.

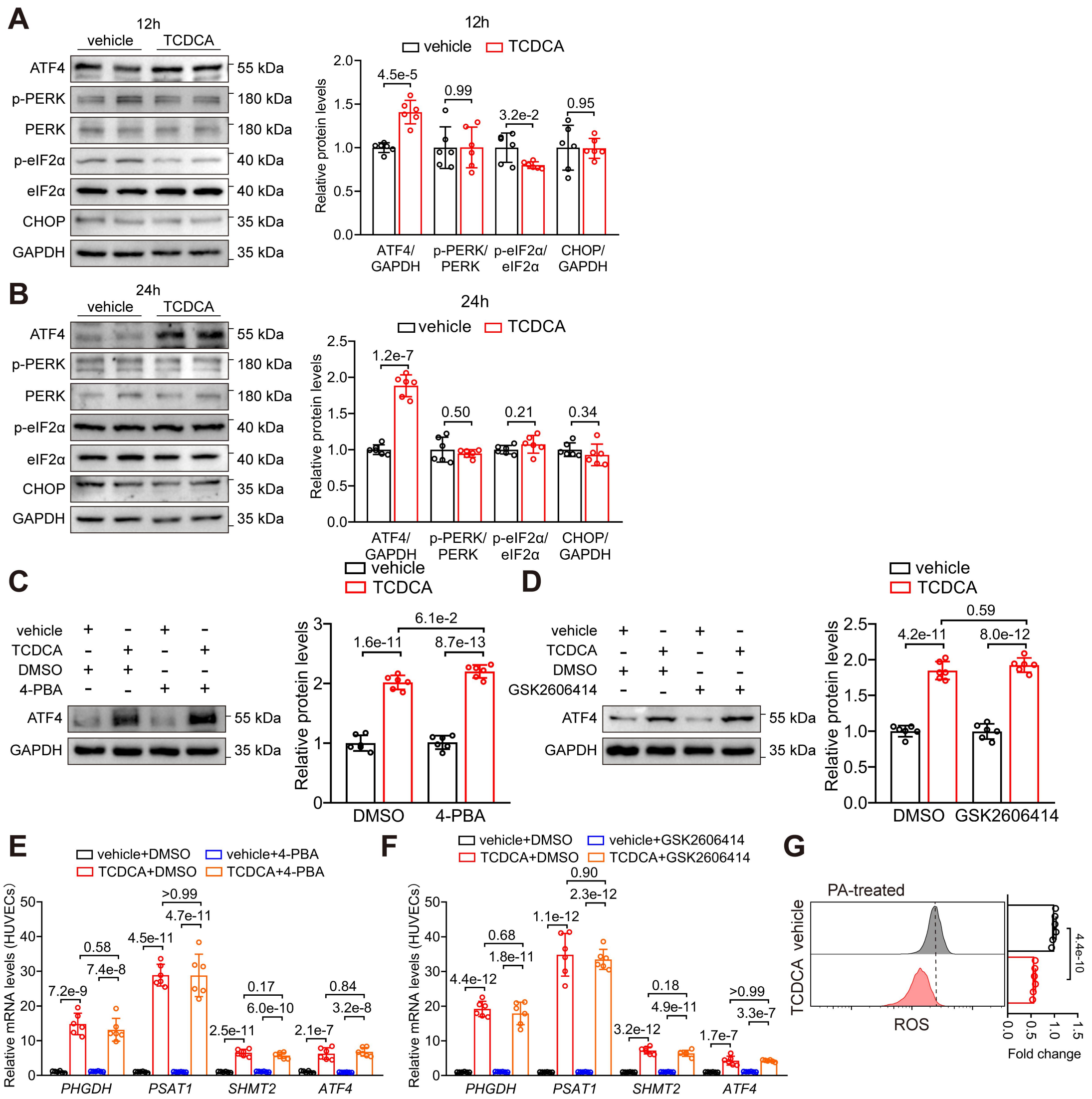

Figure S11 Effect of TCDCA on ATF4 Expression is not Mediated by Endoplasmic Reticulum Stress

(A-B) Representative Western blot (left) and quantification of ATF4/GAPDH, p-PERK/PERK, p-eIF2α/eIF2α and CHOP/GAPDH protein levels (right) in HUVECs treated with vehicle or TCDCA (50 μM, 12 h) or TCDCA (50 μM, 24 h) (n=6).

(C-D) Representative Western blot (left) and quantification of ATF4/GAPDH protein levels (right) in HUVECs treated with vehicle or TCDCA (50 μM, 12 h) after preincubation with 4-PBA (10 μM, C) or GSK2606414 (10 μM, D) (n=6).

(E-F) qPCR analysis of the mRNA levels of *PHGDH*, *PSAT1*, *SHMT2*, and *ATF4* in HUVECs treated with vehicle or TCDCA (50 μM, 12 h) after preincubation with 4-PBA (10 μM, E) or GSK2606414 (10 μM, F) (n=6).

(G) Representative flow cytometric images and analysis of the total ROS levels in HUVECs treated with vehicle or TCDCA (50 μM) normalized to vehicle (n=6).

Data are presented as mean ± SD. Statistical analysis was performed using Student *t* test or Student *t* test with Welch correction (A, B and G) and two-way ANOVA followed by Tukey's post-hoc test (C-F).

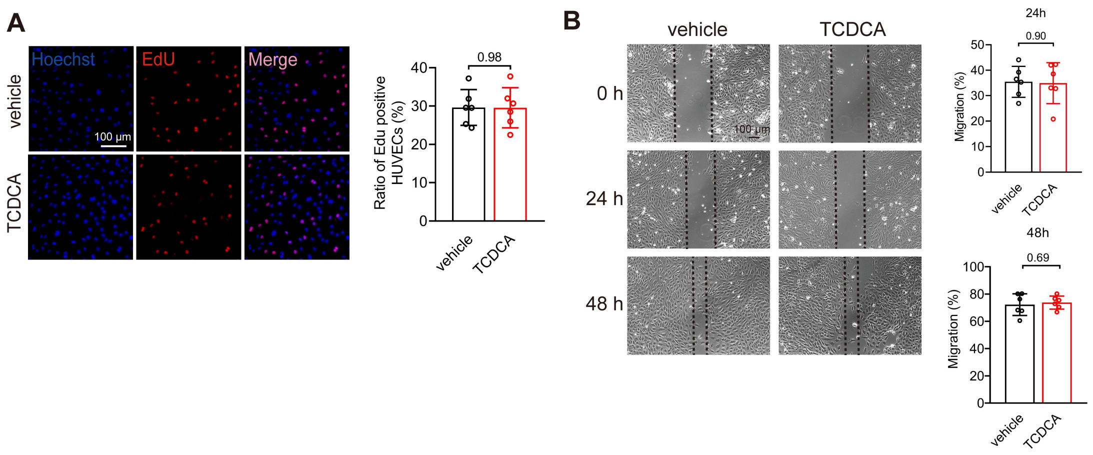

Figure S12 TCDCA's Effects on EC Proliferation and Migration

(A) Representative EdU staining and quantification of the percentage of EdU-positive HUVECs treated with vehicle or TCDCA (50 μM) (n=6).

(B) Representative images and quantification of the scratch assays for 24h or 48h in HUVECs treated with vehicle or TCDCA (50 μM) (n=6).

Data are presented as mean ± SD. Statistical analysis was performed using Student *t* test (A and B).

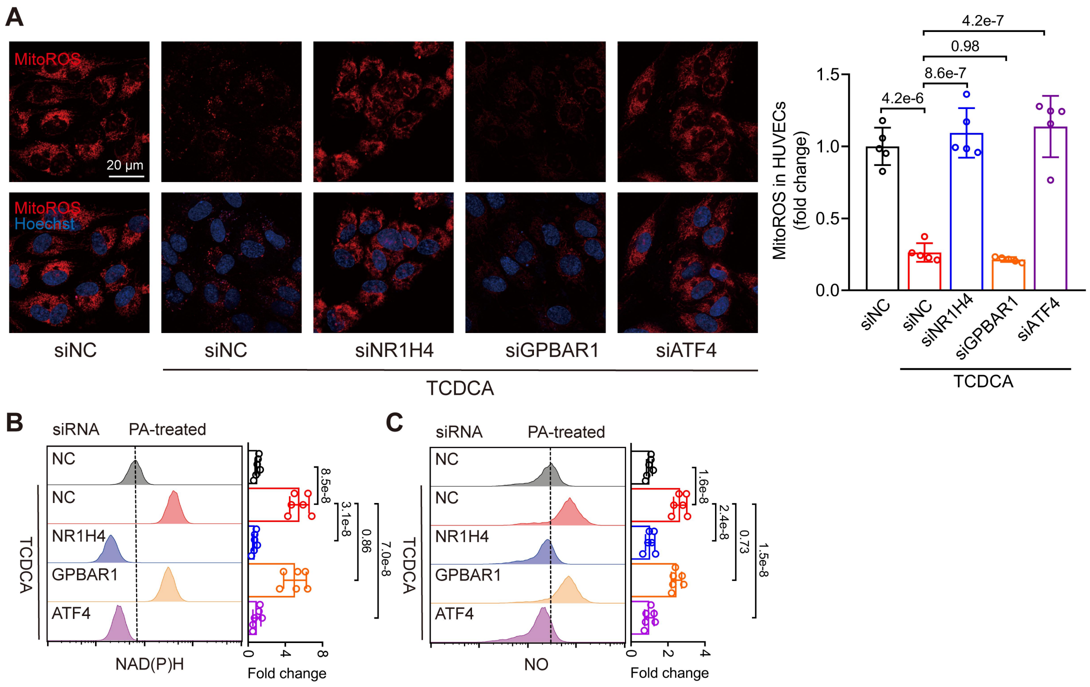

Figure S13 SiRNA Experiments Reveal the Roles of FXR, ATF4, and TGR5

(A) Representative images of mitoROS staining in PA-pretreated (0.3 mM) HUVECs treated with vehicle or TCDCA (50 μM, 24 h) transfected with NC, NR1H4, GPBAR1, or ATF4 siRNA (scale bar = 20 μm), along with quantified mitoROS levels normalized to siNC (n=6).

(B-C) Representative flow cytometric images and analysis of NAD(P)H (B) and NO (C) levels in PA-pretreated (0.3 mM) HUVECs treated with vehicle or TCDCA (50 μM, 24 h) transfected with NC, NR1H4, GPBAR1, or ATF4 siRNA normalized to siNC (n=6).

Data are presented as mean ± SD. Statistical analysis performed using two-way ANOVA followed by Tukey's post-hoc test (A-C).

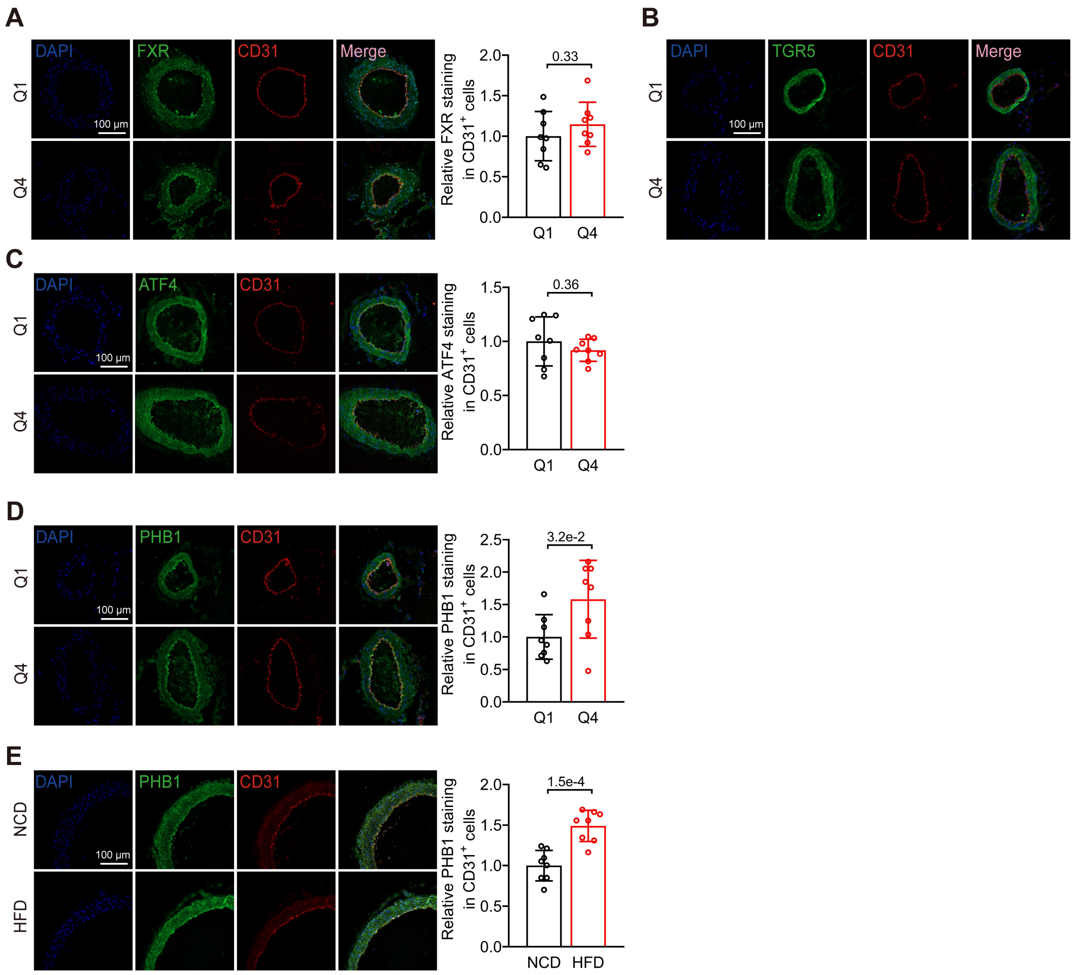

Figure S14 IF staining of FXR, TGR5, ATF4, and PHB1

(A-C) Representative IF staining in arterioles from the Q1 and Q4 groups (scale bar = 100 µm). Panels show staining for: FXR (green) and CD31 (red) in (A); TGR5 (green) and CD31 (red) in (B); ATF4 (green) and CD31 (red) in (C); and PHB1 (green) and CD31 (red) in (D). Quantitative analysis shows fluorescence intensity within $CD31^{+}$ cells for FXR (A), ATF4 (C) and PHB1 (D) (n=8).

(E) Representative PHB1 (green) and CD31 (red) IF staining and quantitative analysis in aortas of normal chow diet (NCD) and high-fat diet (HFD) mice (n=8).

Data are presented as mean ± SD. Statistical analysis was performed using Student *t* test (A, C, D, and E)

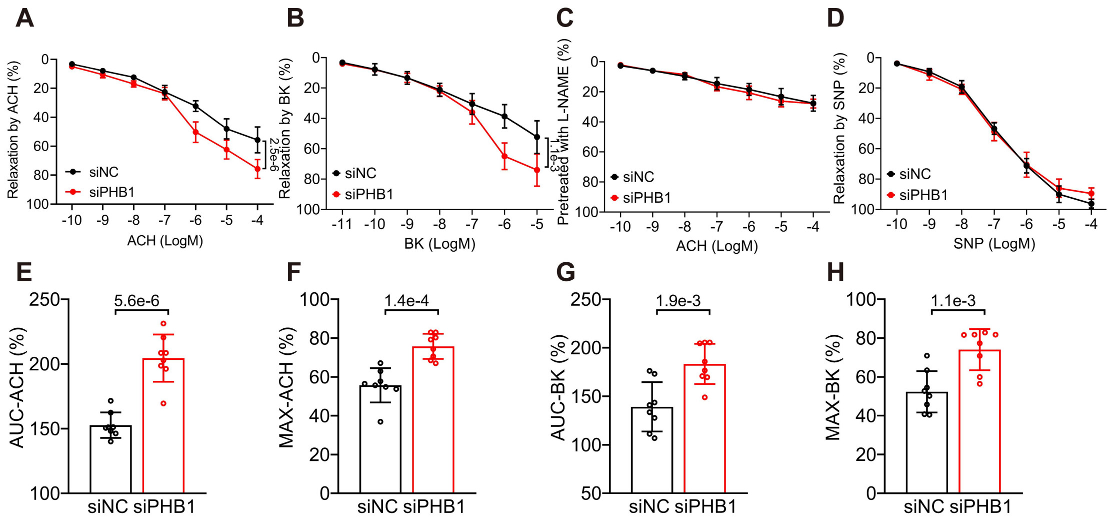

Figure S15 PHB1 Knockdown Aggravates Obesity-Induced ED

(A-B) Endothelium-dependent vasodilation to cumulative concentration of ACH (A) or BK (B) in DIO mice mesenteric arteries transfected with NC or PHB1 siRNA (n=8).

(C-D) Endothelium-independent vasodilation to cumulative concentration of ACH pretreated with L-NAME (C) or to cumulative concentration of SNP (D) in DIO mice mesenteric arteries transfected with NC or PHB1 siRNA (n=8).

(E-H) AUC-ACH (E), MAX-ACH (F), AUC-BK (G), and MAX-BK (H) levels in DIO mice mesenteric arteries transfected with NC or PHB1 siRNA (n=8).

Data are presented as mean ± SD. Statistical analysis was performed using two-way ANOVA followed by Bonferroni's post-hoc test (A and B) and Student *t* test (E-H).

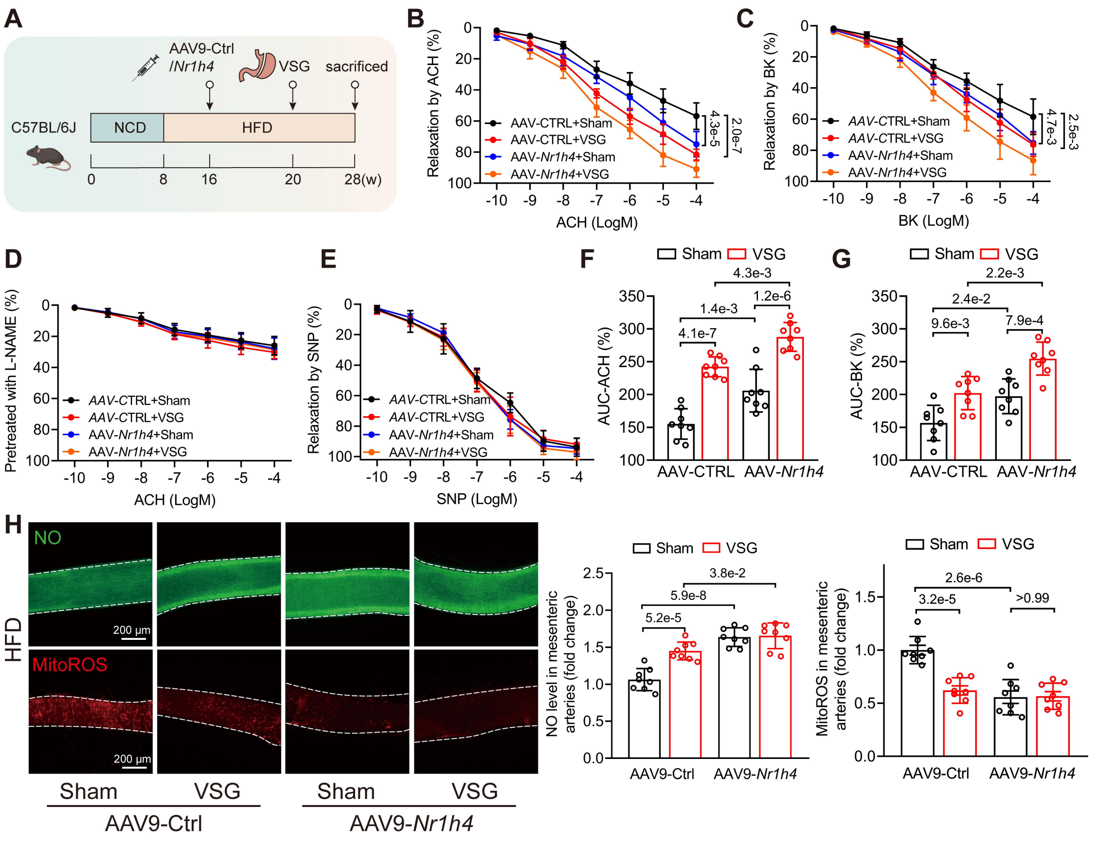

Figure S16 Endothelial FXR Overexpression by AAV9 Alleviating Obesity-induced ED

(A) Schematic diagram of Sham- or VSG-treated DIO mice injected with AAV9-Ctrl or AAV9-*Nr1h4* experiment flow chart.

(B-C) Endothelium-dependent vasodilation to cumulative concentration of ACH (B) or BK (C) in Sham- or VSG-treated DIO mice injected with AAV9-Ctrl or AAV9-*Nr1h4* mesenteric arteries (n=8).

(D-E) Endothelium-independent vasodilation to cumulative concentration of ACH pretreated with L-NAME (D) or to cumulative concentration of SNP (E) in these 4 groups mesenteric arteries (n=8).

(F-G) AUC-ACH (F) and AUC-BK (G) levels in these 4 groups mesenteric arteries (n=8).

(H) Representative images of mitoROS and NO staining in these 4 groups mesenteric arteries (scale bar = 200 μm), along with quantified mitoROS and NO levels (n=8). Data are presented as mean ± SD. Statistical analysis performed using two-way ANOVA followed by Tukey's multiple comparisons test (B, C, F, G, and H).

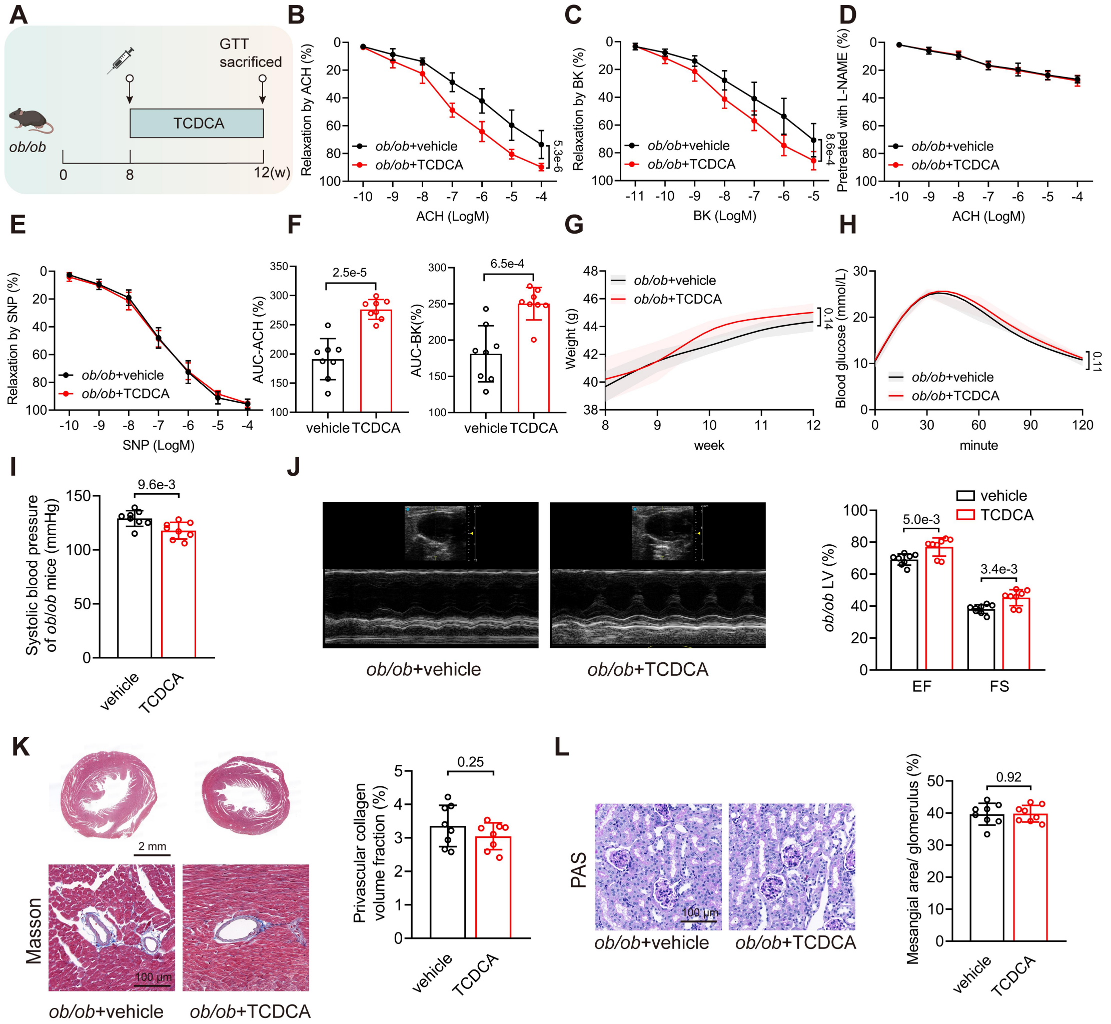

Figure S17 TCDCA Rescues Obesity-induced ED in *ob/ob* mice

(A) Vehicle- or TCDCA-treated *ob/ob* mice experiment flow chart.

(B-C) Endothelium-dependent vasodilation to cumulative concentration of ACH (B) or BK (C) in vehicle- or TCDCA-treated *ob/ob* mice mesenteric arteries (n=8).

(D-E) Endothelium-independent vasodilation to cumulative concentration of ACH pretreated with L-NAME (D) or to cumulative concentration of SNP (E) in vehicle- or TCDCA-treated *ob/ob* mice mesenteric arteries (n=8).

(F) AUC-ACH and AUC-BK levels in vehicle- or TCDCA-treated *ob/ob* mice mesenteric arteries (n=8).

(G-H) Body weight (G) and blood glucose (H) presented as a locally weighted scatterplot smoothing (LOESS) fitting curve in vehicle- or TCDCA-treated *ob/ob* mice (n=8).

(I) Systolic blood pressure (SBP) levels of vehicle- or TCDCA-treated *ob/ob* mice (n=8).

(J) Representative images of echocardiography, ejection fraction (EF) and fractional shortening (FS) in vehicle- or TCDCA-treated *ob/ob* mice (n=8).

(K) Masson staining of the hearts in vehicle- or TCDCA-treated *ob/ob* mice and quantification of perivascular collagen volume fraction (n=8).

(L) PAS staining of the kidneys in vehicle- or TCDCA-treated *ob/ob* mice and quantification of mesangial area/glomerulus (n=8).

Data are presented as mean ± SD. Statistical analysis performed using two-way ANOVA followed by Bonferroni's post-hoc test (B, C, G, and H) and Student t test (F, I, J, K and L).

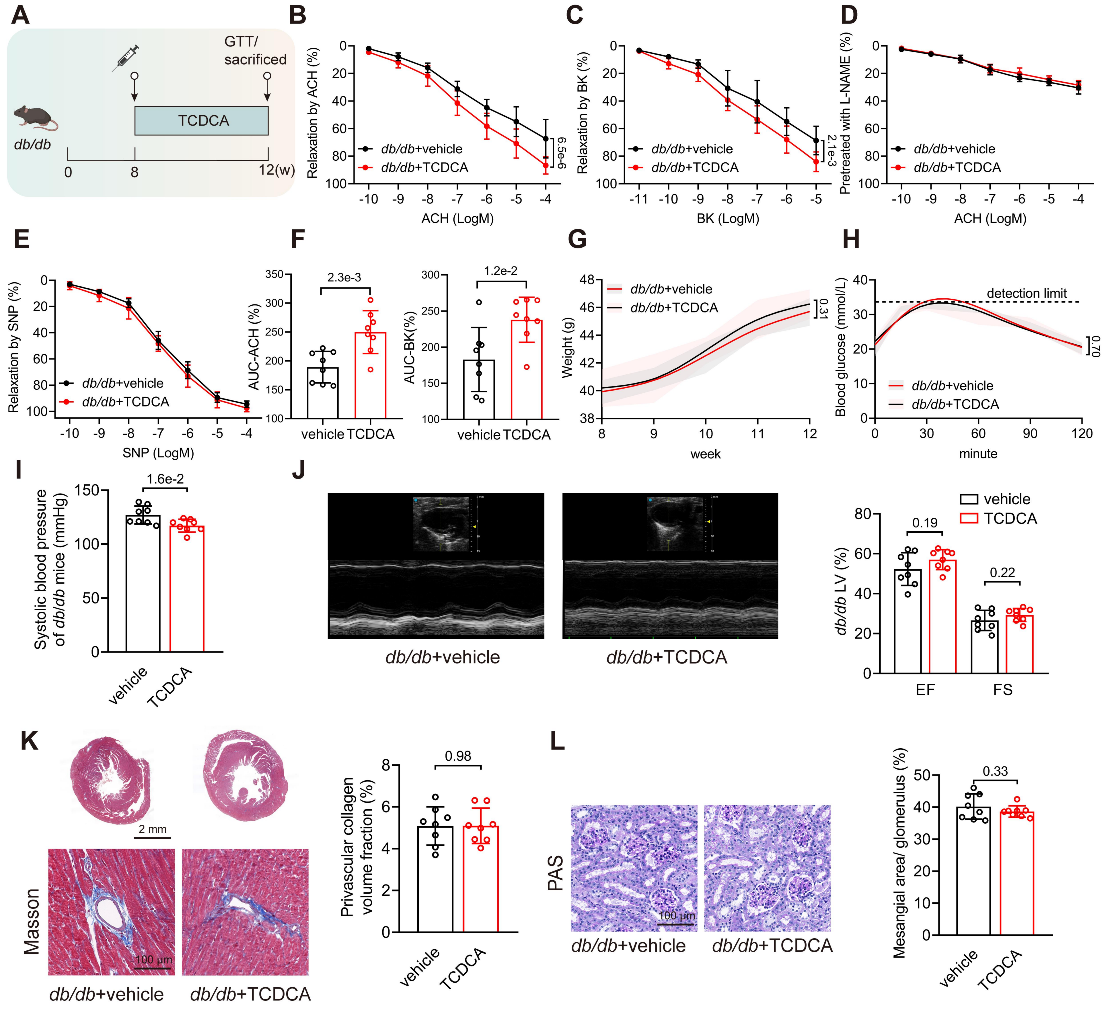

Figure S18 TCDCA Rescues Obesity-induced ED in *db/db* mice

(A) Vehicle- or TCDCA-treated *db/db* mice experiment flow chart.

(B-C) Endothelium-dependent vasodilation to cumulative concentration of ACH (B) or BK (C) in vehicle- or TCDCA-treated *db/db* mice mesenteric arteries (n = 8).

(D-E) Endothelium-independent vasodilation to cumulative concentration of ACH pretreated with L-NAME (D) or to cumulative concentration of SNP (E) in vehicle- or TCDCA-treated *db/db* mice mesenteric arteries (n = 8).

(F) AUC-ACH and AUC-BK levels in vehicle- or TCDCA-treated *db/db* mice mesenteric arteries (n = 8).

(G-H) Body weight (G) and blood glucose (H) presented as a locally weighted scatterplot smoothing (LOESS) fitting curve in vehicle- or TCDCA-treated *db/db* mice (n=8).

(I) Systolic blood pressure (SBP) levels of vehicle- or TCDCA-treated *db/db* mice (n=8).

(J) Representative images of echocardiography, ejection fraction (EF) and fractional shortening (FS) in vehicle- or TCDCA-treated *db/db* mice (n=8).

(K) Masson staining of the hearts in vehicle- or TCDCA-treated *db/db* mice and quantification of perivascular collagen volume fraction (n=8).

(L) PAS staining of the kidneys in vehicle- or TCDCA-treated *db/db* mice and quantification of mesangial area/glomerulus (n=8).

Data are presented as mean ± SD. Statistical analysis performed using two-way ANOVA followed by Bonferroni's post-hoc test (B, C, G, and H) and Student *t* test (F, I, J, K and L).

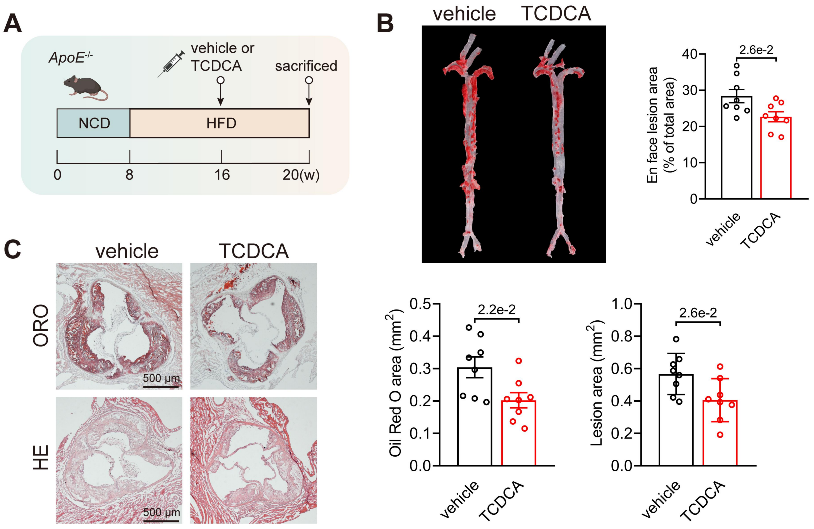

Figure S19 TCDCA ameliorates the development of atherosclerosis

(A) Vehicle- or TCDCA-treated *ApoE*$^{-/-}$ mice experiment flow chart.

(B) Representative oil red O (ORO) staining (left) and *en face* analysis of atherosclerotic lesions (right) in the whole aorta (n=8).

(C) ORO staining and cross-sectional analysis of atherosclerotic lesions in the aortic root. Hematoxylin and eosin (HE) staining and cross-sectional analysis of atherosclerotic lesions in the aortic root (n=8).

Data are presented as mean ± SD. Statistical analysis performed using Student *t* test (B and C).

Table S1. Characteristics of the NHO patients (n = 213)

Characteristics	MHO (n = 62)	MUO (n = 151)	*P*
Age, y	32.00 (26.00, 36.50)	33.00 (28.00, 39.00)	0.247
Male/female	27/35	74/77	0.469
Height, cm	168.00 (163.00, 176.50)	172.00 (165.00, 178.00)	0.146
Weight, kg	120.00 (100.00, 140.00)	122.20 (98.60, 141.50)	0.744
BMI, kg/m^2	40.52 (37.45, 46.88)	40.50 (36.46, 46.75)	0.676
WAIST, cm	124.50 (113.00, 140.00)	127.00 (115.00, 140.00)	0.789
BFP, %	42.75 (40.10, 44.80)	39.80 (36.90, 43.20)	0.001
VAT area, cm^2	193.00 (161.00, 224.00)	190.00 (160.00, 229.50)	0.954
SBP, mmHg	132.00 (123.00, 136.00)	129.00 (125.00, 132.00)	0.445
DBP, mmHg	85.00 (76.00, 88.00)	85.00 (79.00, 87.00)	0.623
Current smoking	7 (11.29%)	21 (13.91%)	0.608
History of alcoholism	8 (12.90%)	11 (7.28%)	0.197
HDL-C, mmol/L	1.29 (1.14, 1.45)	0.98 (0.85, 1.12)	0.000
FFA, mmol/L	62.05 (50.00, 82.00)	67.80 (55.40, 83.25)	0.147
FBG, mmol/L	4.95 (4.47, 5.35)	5.66 (4.80, 7.43)	0.000
AST, U/L	25.00 (17.00, 43.00)	24.00 (18.00, 39.50)	0.609
ALT, U/L	26.00 (17.00, 43.00)	30.00 (20.00 61.50)	0.068
TG, mmol/L	1.32 (1.00, 1.58)	2.01 (1.41, 2.72)	0.000
UA, μmol/L	426.50 (347.00, 516.00)	412.00 (340.00, 485.50)	0.372
BUN, mmol/L	4.70 (3.90, 5.60)	4.70 (4.15, 5.70)	0.524
CR, μmol/L	62.50 (54.00, 69.00)	58.00 (50.50, 69.00)	0.197

Data are presented as median (interquartile range) for continuous data and number (percentage) for catagorical data. BMI, body mass index; SBP, systolic blood pressure; DBP, diastolic blood pressure; HDL-C, high-density lipoprotein cholesterol; FFA, free fatty acids; FBG, fasting blood glucose; AST, aspartate aminotransferase; ALT, alanine aminotransferase; TG, triglyceride; UA, uric acid; BUN, blood urea nitrogen; CR, creatinine; BFP, body fat percentage; VAT, visceral adipose tissue.

Table S2. Interaction analysis with group (MHO vs MUO)

Characteristics	*P* for interaction
BMI, kg/m^2	0.275
WAIST, cm	0.125
BFP, %	0.244
VAT area, cm^2	0.786
SBP, mmHg	0.062
DBP, mmHg	0.658
HDL-C, mmol/L	0.246
FFA, mmol/L	0.304
FBG, mmol/L	0.566
AST, U/L	0.596
ALT, U/L	0.842
TG, mmol/L	0.940
UA, μmol/L	0.041
BUN, mmol/L	0.149
CR, μmol/L	0.111

Table S3. Characteristics of the NHO patients in Figure 3 (n = 33)

Characteristics	MHO (n = 13)	MUO (n = 20)	*P*
Age, y	31.00 (25.50, 35.50)	29.50 (26.25, 39.50)	0.730
Male/female	3/10	4/16	0.045
Height, cm	164.00 (159.50, 177.00)	165.00 (162.25, 170.00)	0.624
Weight, kg	100.00 (94.40, 130.00)	101.50 (93.50, 109.75)	0.703
BMI, kg/m^2	38.57 (35.28, 43.00)	36.73 (34.93, 38.95)	0.164
WAIST, cm	120.00 (111.00, 131.00)	112.00 (104.50, 118.25)	0.110
BFP, %	42.20 (40.25, 45.00)	41.55 (40.05, 42.95)	0.298
VAT area, cm^2	179.00 (147.00, 220.50)	175.50 (146.25, 203.75)	0.421
SBP, mmHg	124.00 (114.50, 128.00)	128.50 (119.00, 133.00)	0.147
DBP, mmHg	81.00 (76.00, 82.00)	77.00 (72.25, 83.50)	0.353
HDL-C, mmol/L	1.25 (1.15, 1.33)	0.98 (0.90, 1.05)	0.000
FFA, mmol/L	70.90 (55.00, 89.30)	62.50 (49.40, 71.50)	0.188
FBG, mmol/L	4.77 (4.21, 5.22)	6.15 (5.34, 8.34)	0.002
AST, U/L	28.00 (17.00, 36.50)	21.50 (19.00, 35.00)	0.624
ALT, U/L	37.00 (21.00, 65.50)	31.50 (18.75 54.00)	0.548
TG, mmol/L	1.05 (0.81, 1.61)	1.64 (1.26, 1.98)	0.048
UA, μmol/L	360.00 (291.50, 440.50)	400.00 (348.50, 450.75)	0.257
BUN, mmol/L	4.40 (3.33, 5.15)	4.25 (3.47, 5.40)	0.813
CR, μmol/L	58.00 (51.00, 69.00)	58.50 (48.00, 63.00)	0.318

Data are presented as median (interquartile range). BMI, body mass index; SBP, systolic blood pressure; DBP, diastolic blood pressure; HDL-C, high-density lipoprotein cholesterol; FFA, free fatty acids; FBG, fasting blood glucose; AST, aspartate aminotransferase; ALT, alanine aminotransferase; TG, triglyceride; UA, uric acid; BUN, blood urea nitrogen; CR, creatinine; BFP, body fat percentage; VAT, visceral adipose tissue.

Table S4. Primer sequences used in this study

Gene	Forward	Reverse
Homo-*PHGDH*	ATCTCTCACGGGGGTTGTG	AGGCTCGCATCAGTGTCC
Homo-*PSAT1*	ACTTCCTGTCCAAGCCAGTGGA	CTGCACCTTGTATTCCAGGACC
Homo-*SHMT2*	GCCTCATTGACTACAACCAGCTG	ATGTCTGCCAGCAGGTGTGCTT
Homo-*ATF4*	GTCCTCCACTCCAGATCATTC	AGTCTGGCTTCCTATCTCCTTC
Homo-*MTHFD2*	TTCTGGAAGGAAACTGGCCC	TTCTCGCCAACCAGGATCAC
Homo-*ALDH1L2*	GCTGAAGTTGGCACTAATTGGC	TGAACACCCCTACTACTCGGT
Homo-*PSPH*	TTGATGAGACGCAGCCAACA	GCAGGAGGACAGGCTTCCAT
Homo-*PCK2*	GAAAACCCTGATTGGCCACG	GATGCCCAGGATCAGCATGT
1 ChIP for *ATF4*	AGATATATATGATTCACCGGGCC	TTATTGTGAAGGCCGAGGAC
2 ChIP for *ATF4*	CGTCCTCGGCCTTCACAATA	GAGTCCGTTCTTTCCTTCGC

Table S5. siRNA sequences used in this study

Gene	Sense	Antisense
Homo-*NR1H4*	GUGGUACUCUCCUGGAAUATT	UAUUCCAGGAGAGUACCACTT
Homo- *GPBAR1*	GCAAAGACGAGUCAAGUUUTT	AAACUUGACUCGUCUUUGCTT
Homo-*PHB1*	GGACUUGUAUAGUGAGAGATT	UCUCUCACUAUACAAGUCCTT
Homo-*PHB2*	GCUCCAAAGACCUACAGAUTT	AUCUGUAGGUCUUUGGAGCTT
Homo-*ATF4*	GACAAAGACACCUUCGAAUTT	AUUCGAAGGUGUCUUUGUCTT

References

1. Xie G, Wang L, Chen T, Zhou K, Zhang Z, Li J, et al. A Metabolite Array Technology for Precision Medicine. *Anal Chem* 2021;**93**:5709-5717. doi: https://doi.org/10.1021/acs.analchem.0c04686
